# Deciphering the Natural Reassortment Dynamics of Infectious Bursal Disease Virus, Isolated from Field Outbreaks in Southern India, Through Complete Genome Sequencing

**DOI:** 10.3390/pathogens15010026

**Published:** 2025-12-24

**Authors:** Raja Paramasivam, Megan Justice, Tuticorin Maragatham Alagesan Senthilkumar, Manoharan Parthiban, Ardhanary Thangavelu, Angappan Mangala Gowri, Ramasamy Bharathi, Hong Hwang, Jerry Malayer, Samuel Pushparaj

**Affiliations:** 1 Department of Animal Biotechnology, Madras Veterinary College, Tamil Nadu Veterinary and Animal Sciences University, Chennai 600 007, India; drparthiban66@gmail.com; 2 Next Generation Sequencing Core Facility, Oklahoma State University, Stillwater, OK 74075, USA; megan.justice@okstate.edu (M.J.); hongjin.hwang@okstate.edu (H.H.); 3 Department of Veterinary Microbiology, Madras Veterinary College, Tamil Nadu Veterinary and Animal Sciences University, Chennai 600 007, India; thangavelu_a@hotmail.com; 4 Centralized Instrumentation Laboratory, Madras Veterinary College, Tamil Nadu Veterinary and Animal Sciences University, Chennai 600 007, India; gowrivalavan1@gmail.com; 5 Central University Laboratory, Tamil Nadu Veterinary and Animal Sciences University, Chennai 600 051, India; rbdhasan@gmail.com; 6 Department of Physiological Sciences, Oklahoma State University, Stillwater, OK 74075, USA; jerry.malayer@okstate.edu

**Keywords:** infectious bursal disease virus, complete genome sequencing, reassortment, India, recombination, genetic diversity, phylogenetic analysis

## Abstract

The present study was carried out to analyze the complete genome sequences of infectious bursal disease virus (IBDV) isolates obtained from field outbreaks in the southern regions of India. Bursal tissue samples were collected and screened by RT-PCR, targeting the VP2 gene. Positive samples were subjected to serological identification via AGID. Following this, eight samples (BGE14, BGE15, MDI14, THI14, EDE14, RPM14, VCN14, and NKL14) were subjected to virus isolation in 9 to 11-day-old embryonated chicken eggs, and their complete genomes were sequenced. Analysis of the VP2 hypervariable region (HVR) revealed that all eight isolates had five unique and highly conserved amino acids (A222, I242, Q249, I256, and S299). However, all the isolates reveal a substitution of Isoleucine by Valine at residue 294 (I294V). Furthermore, analysis of segment B from all Indian IBDV sequences revealed that the triplet amino acid pattern was NEG (residues 145–147) and the amino acid at position 242 was consistently D across all isolates. These findings suggest that segment B of the isolates in this study resembled that of vaccine strains and non-vvIBDV strains. Additionally, the presence of the signature D242 in all Indian isolates, characteristic of non-vvIBDV strains, implies a potential attenuation. Moreover, in the phylogenetic analysis of VP2-HVR, all isolates clustered with very virulent reference strains, while segment B clustered with classical attenuated strains. Notably, the phylogenetic analysis of VP2-HVR and VP1 of these viruses demonstrated genetic variances, suggesting evolutionary changes in segment B across all eight Indian isolates, likely indicative of natural genome reassortment resulting in these specific outbreaks in the flocks.

## 1. Introduction

The poultry industry in India is one of the fastest growing agricultural sub-sectors, contributing significantly to the country’s food security, employment opportunities, and economic growth. The major problems threatening the poultry industry, such as low weight gain, low egg production, low meat production, and heavy economic losses, are due to nutrition, managemental practices, various infectious diseases, and vaccination failures [1]. Among them, infectious bursal disease virus (IBDV) is a highly contagious birnavirus responsible for inducing severe immunosuppressive conditions in chickens. IBDV exhibits notable antigenic, pathogenic, and genetic diversity, which profoundly influences disease prevention, control strategies, and diagnostic techniques [2]. It is one of the deadliest immunosuppressive viral diseases in poultry, which may, for undetermined reasons, affect the flock even after the administration of vaccines.

Infectious bursal disease virus, originally reported in the 1960s in Gumboro, Delaware (USA), has since disseminated globally, impacting poultry industries across Asia, Europe, Africa, and the Americas. From its initial emergence, infectious bursal disease (IBD) has remained endemic in numerous regions, continuing to pose a significant and ongoing threat to poultry health worldwide. The disease persisted in its classical form until the early 1990s, with mortality rates reaching up to 30%. However, during the late 1990s, significantly higher mortality associated with IBD was reported. Despite adherence to recommended vaccination schedules, the disease continued to cause substantial economic losses to poultry farmers worldwide, primarily due to its immunosuppressive nature [3,4,5,6]. The disease may be fatal when highly virulent strains are involved. Several antigenic variants of IBDV have been identified, and these strains often cause subclinical infections that result in varying levels of immunosuppression. As the immune system becomes compromised, affected birds exhibit increased susceptibility to other pathogens, including Newcastle disease virus (NDV), infectious bronchitis virus (IBV), infectious laryngotracheitis virus (ILT), *Salmonella* spp., and opportunistic bacteria such as *Escherichia coli*. In addition, immunosuppressed birds generally show poor vaccine responsiveness and may experience pronounced or prolonged post-vaccination reactions [7].

Two IBDV serotypes, namely serotype I and serotype II, have been identified [8], with subtypes based on the antigenic variation within each serotype [9]. Both serotypes naturally infect chickens and turkeys, but only serotype I is pathogenic to chickens, causing clinical disease in the birds [10,11]. Based on virulence and antigenicity, serotype I viruses have been classified as attenuated, intermediate virulent, classical virulent, variant, and very virulent [4,12]. Currently, serotype I IBDV viruses are antigenically grouped as classic (also known as standard) and variant strains based on a virus neutralization assay [13]. The very virulent IBDV (vvIBDV) strains are considered to be the most antigenically similar to classic IBDV strains, despite their enhanced pathogenic properties and ability to penetrate maternal immunity induced by classical, mild IBDV vaccines [14]. These findings suggest that vvIBDVs are continuously evolving in nature, and further investigation is needed to determine the biological significance of these antigenic differences [15,16,17].

The IBD genome consists of two segments, namely segment A and B. Segment A encodes a polyprotein (VP2–VP4–VP3) that is cleaved into structural proteins, along with VP5, a non-structural protein involved in virus release. VP2 forms the outer capsid and is the main antigenic protein, while VP3 and VP4 play roles in capsid stability and polyprotein processing, respectively. Segment B encodes VP1, the RNA-dependent RNA polymerase essential for viral replication. Together, these proteins contribute to viral assembly, pathogenesis, and immune response. Molecular characterization of IBDV has been based mainly on the study of the VP2 gene, the middle third of which contains a variable region where stretches of hydrophilic amino acids have been recognized as the molecular basis for antigenic variation [18,19,20]. Several amino acids in VP2 have also been proposed as putative markers for pathogenic IBDV. The amino acids present in the VP2 region of vvIBDV isolates have revealed four conserved residues at A222, I256, I294, and S299 as compared to classical IBDV isolates [21,22]. These positions have been used as the target for several molecular or antigenic tests aimed at presumptive molecular or antigenic identification of vvIBDVs [23,24]. By using the reverse-genetic system developed for IBDV [25], it was demonstrated that VP2 mutations Q253H, D279N, and A284T are involved in both cell culture adaptation and attenuation [26,27,28,29]. Although VP5, encoded by segment A, has been implicated in virulence in a limited number of strains, some studies suggest that segment B of vvIBDV strains may also contribute to pathogenicity and exhibit distinct genetic features. Studies using laboratory-generated chimeric viruses have demonstrated that segment B contributes to viral replication efficiency [30,31,32]. Together, these observations indicate that both segment A and segment B play roles in determining IBDV pathogenicity [33].

Many reports ascertain that major changes in virulence could be the result of just one or two base changes in coding or non-coding sequences. These mutations may be in the VP4 viral protease, in VP1 polymerase, near the VP2–VP4 cleavage site, and/or in the antigenic sites of VP2 and VP3. Among the structural proteins, VP2 contains important neutralizing antigenic sites and elicits protective immune response [34]. Most of the amino acid (aa) changes between antigenically different IBDVs are clustered in the hypervariable region of VP2. Since this hypervariable region exhibits various distinct mutations depending on the IBDV strain, most of the molecular techniques for genotyping IBDV have traditionally focused on analyzing this region. As a result, other parts of the IBDV genome, such as segment B, have traditionally been understudied.

In recent years, molecular classification of infectious bursal disease virus (IBDV) has shifted from traditional pathotype-based categories (classic, variant, and very virulent strains) toward phylogeny-driven genotyping schemes that incorporate both genome segments. Earlier systems relied exclusively on the hypervariable region of VP2 (hvVP2), but these approaches could not identify segment reassortment events. To address this, Wang et al. [35] proposed a unified bisegment genotyping system that assigns each genome segment to the distinct genogroups A0–A8 for segment A and B1–B5 for segment B, allowing clear designation of strain genotypes as A*XB*X combinations and enabling detection of natural reassortants. This concept was further refined by [36], who recommended integrating hvVP2 (segment A) and partial VP1 (segment B) phylogenies to improve resolution of emerging variant and vvIBDV-like strains. More recently, global surveillance studies have identified additional genogroups, including A2d and B1b, associated with the rapid intercontinental spread of Chinese “novel variant” IBDV, now reported across Asia, Africa, and South America [37]. Collectively, these updated classification schemes provide a more robust, evolution-informed framework for tracking IBDV diversity, reassortment patterns, and the emergence of region-specific viral lineages.

The present study presents the complete genome sequences of Indian isolates of IBDV, which may reveal the exact nature of virulence with which they are associated. Determination of the complete sequence of IBDV is a prerequisite for identifying the determinants responsible for its virulence and would facilitate the production of specific sero-diagnostic tools for the discrimination of vvIBDV from other pathotypes. It is expected that the findings of this study will contribute to the knowledge of the current IBDV strains circulating in the southern states of India.

## 2. Materials and Methods

### 2.1. Sample Collection and Processing

**Figure 1:**
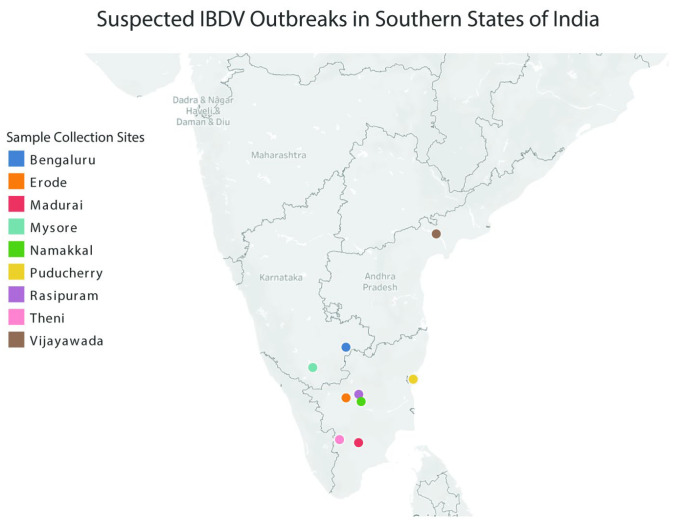
Map of southern India highlighting suspected IBDV outbreak sites where the field isolates evaluated in this publication were collected.

A total of 113 samples were collected from nine outbreak farms across the southern Indian states. Birds were selected based on field observations indicative of suspected IBDV infection, such as sudden-onset mortality, depression, ruffled feathers, watery diarrhea, and characteristic bursal enlargement or hemorrhage (Figure 1). The agar gel immunodiffusion test (AGID) was carried out to confirm the IBDV presence. Suspension of the tissues was prepared with phosphate-buffered saline (SRL, Milan, Italy) (1 mL) of pH 7.4, centrifuged for 30 min at 2000× *g*, and stored at −80 °C.

### 2.2. Propagation of IBDV In Vitro

The samples BGE14, BGE15, MDI14, THI14, EDE14, RPM14, VCN14, and NKL14 collected from 3–5 week old chickens of various IBD outbreaks that were confirmed to be positive for IBDV were selected for virus isolation. Tissue samples were homogenized in sterile phosphate-buffered saline (PBS) containing a standard antibiotic–antimycotic mixture (penicillin 100 IU/mL, streptomycin 100 µg/mL, amphotericin-B 0.25 µg/mL) was obtained from Gibco (Thermo Fisher Scientific, Waltham, MA, USA) and clarified by low-speed centrifugation using an Eppendorf 5810 R centrifuge (Eppendorf SE, Hamburg, Germany) at 3000× *g* for 10 min. The supernatant was further filtered through a 0.45 μm syringe filter (Merck KGaA, Darmstadt, Germany) to remove debris. Approximately 0.1 mL of the prepared inoculum was inoculated onto the chorioallantoic membrane (CAM) route. The CAM was harvested in the following days and stored at −80 °C.

### 2.3. RNA Isolation and RT-PCR

RNA extraction was performed with the bursal tissue homogenate in PBS by the conventional Trizol method. A total of 250 µL of the tissue lysate was added to 750 µL of Trizol (Takara Bio, Shiga, Japan) and 150 µL of chloroform (Merck KGaA, Darmstadt, Germany). The aqueous layer was collected after centrifugation at 10,000 rpm for 10 min. An equal volume of ice-cold isopropanol (Merck KGaA, Darmstadt, Germany) was added to the aqueous layer and left overnight at −80 °C. Later, RNA was precipitated by centrifuging at 12,000 rpm for 20 min, followed by 70% ethanol (Merck KGaA (Darmstadt, Germany)) wash. The RNA pellet was resuspended in 10 µL nuclease-free water and stored at −80 °C. cDNA synthesis was carried out using a cDNA kit (Thermo Scientific, Waltham, MA, USA). PCR was performed against the VP2 region with the primers FP-(5′-GCCCAGAGTCTACACCAT-3′) and 743-RP-(5′-CCCGGATTATGTCTTTGA-3′) for 30 cycles [38]. The primers were used at 20 pmol concentration, and discrete negative and positive control were used.

### 2.4. Complete Genome Amplification of IBDV

The genomic segment A was amplified using three sets of overlapping primers, IBDVSegA 55FP/RP, IBDVSegA 1183FP/RP, and IBDVSegA 1507FP/RP, with expected product sizes of 1539 bp, 983 bp, and 1719 bp, respectively. For sequencing purposes, two additional internal sequencing primers were designed as IBDVSegA 320FP/RP and IBDVSegA 2223FP/RP, with expected product sizes of 520 bp and 513 bp, respectively. The 5′ and 3′ end of segment A was amplified using two sets of primers, IBDVSegA 5′FP/RP and IBDVSegA 3′FP/RP, for the coverage of the extreme 5′ and 3′ ends, with 410 bp and 198 bp, respectively. The genomic segment B was amplified using five sets of overlapping primers, IBDVSegB 40FP/RP, IBDVSegB 651FP/RP, IBDVSegB 1112FP/RP, IBDVSegB 1639FP/RP, and IBDVSegB 2211FP/RP, with expected product sizes of 683 bp, 637 bp, 753 bp, 702 bp, and 587 bp, respectively. The 5′ and 3′ end of segment B was amplified using a set of primers (IBDVSegB 5′FP/RP and IBDVSegB 3′FP/RP) for the coverage of extreme 5′ and 3′ ends, with 169 bp and 224 bp, respectively. The amplification was carried out in 50 µL reaction volume as described earlier; RT-PCR primer details, time, and temperature combinations are given in Supplementary File S1.

### 2.5. Sequence Analysis

The PCR products were sent for sequencing at Messrs, Eurofins Sequencing Services, Bangalore, India, after purification of the product using a QIAquick Gel Extraction Kit (Qiagen, Hilden, Germany). The obtained sequences were blast analyzed, and the consensus sequence was derived using Lasergene software (version 7.1.0). Multiple sequence alignment was performed to understand the amino acid sequences, and the phylogenetic tree was constructed for the VP2 region and segments A and B of the complete genome using the Maximum likelihood method with 1000 bootstrap replications, using the MEGA software (version 11).

### 2.6. Antigenic Index

The Protean program (DNASTAR Lasergene v18.x, DNA Star Inc., Madison, WI, USA) was used to analyze the antigenic sites of segments A and B from the obtained amino acid sequences based on surface probability, regional backbone flexibility, and probable secondary structures, as specified by Jameson and Wolf [39].

### 2.7. Recombination Analysis

The recombination analysis was performed on the complete gene sequences using the Recombination Detection Program (version 4). All the complete segment A and B sequences of IBDV from current studies and complete reference sequences retrieved from Genbank were aligned using CLUSTAL W. The aligned sequences were analyzed with the help of the SimPlot program [40]. Using a similarity plot, Bootscanning plot, and Findsite sub-program, recombination events were detected. The recombination breakpoints were analyzed using a maximization of X2 [41] using SimPlot combined with genetic algorithms using GARD (http://www.datamonkey.org/GARD, accessed on 24 April 2024) [42]. Recombination analysis was performed using segment A and segment B separately, as dsRNA birnaviruses replicate their genome segments independently and concatenation may create artefactual breakpoint signals. For each segment, multiple sequence alignments were generated using MAFFT and were manually inspected to remove ambiguities and ensure codon alignment. These curated alignments were then used as independent inputs in RDP5. All seven algorithms (RDP, GENECONV, Chimaera, MaxChi, BootScan, SiScan, and 3Seq) were run under default parameters, and putative recombination events were considered only when detected by at least five methods, with *p* < 0.05 after Bonferroni correction.

## 3. Results

### 3.1. Field Isolation of IBDV

Bursal tissue samples suspected for IBDV infection were collected from nine different potential outbreak sites in the Southern states of India (Figure 1). Eight bursal homogenates (BGE15, BGE14, VCN14, NKL14, RPM14, THI14, MDI14, and EDE14) from outbreaks confirmed to be positive for IBDV infection by RT-PCR were subjected to virus isolation in embryonated chicken eggs, and complete genome sequencing was carried out. In embryonated chicken eggs, very distinct lesions were observed as early as the third passage onwards. Mortality was observed in inoculated embryos between four to six days post inoculation. The lesions observed in both dead and live embryos were dwarfism, abdominal and cerebral edema, greenish discoloration and ecchymotic hemorrhages of the liver, and necrosis of the kidney. The CAM was thickened and congested, and small opaque pocks were occasionally observed in some of the embryos.

### 3.2. Complete Genome Sequence Analysis of IBDV

#### 3.2.1. Segment A Nucleotide Analysis

To better understand the genetic makeup of IBDV field isolates, we conducted complete genome sequencing of the eight isolates of IBDV (both segment A and segment B). Complete genome sequencing also revealed that the complete nucleotide length of genome segment A in our IBDV isolates varies from 3259 to 3261 bp. Details of the nucleotide length and ORF positions within segment A are shown in Table 1. Multiple alignment of the eight isolates revealed that the predicted aa sequence of the VPX protein (precursor of VP2) consisted of 512 aa, VP4 consisted of 243 aa, and VP3 consisted of 257 aa. Details of aa variation in segment A, including the total number of variants detected, are shown in Table 2.

The starting site of the VP2 is at amino acid 1 from ORF2 and the VX–VP4–VP3 polyprotein of segment A. The VX VP4 cleavage site is at amino acid 512–513, and the VP2 processing site is at amino acid 442–512.

**Figure 2:**
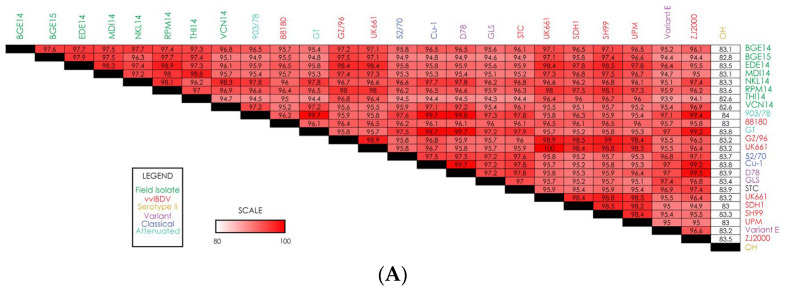

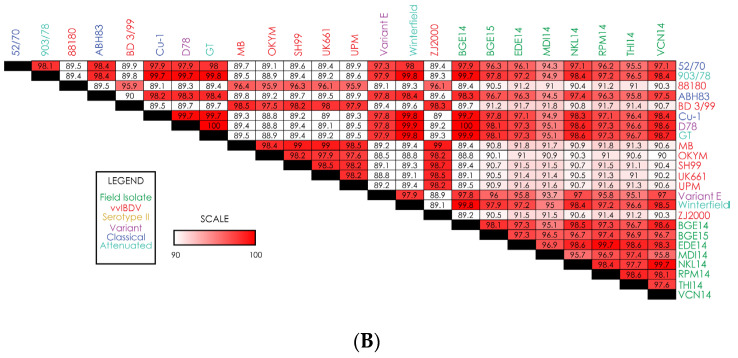
Heatmaps displaying nucleotide-level similarity scores for (**A**) complete genome segment A; (**B**) complete genome segment B. Strain names are color-coded based on IBDV strain type.

We next compared the IBDV field isolate nucleotide sequences to each other as well as to various IBDV reference strains (Figure 2). First, to assess the conservation of segment A among our IBDV field isolates, we compared the nucleotide identity of the segment between all eight isolates (Figure 2A). Many of our field isolates show a high degree of sequence conservation in segment A, with the degree of similarity between many of the isolates being >97%. Interestingly, it seems that isolate VCN14 shows a slightly lower nucleotide similarity the other field isolates, indicating it may be the most diverse or unique isolate we collected. Next, we compared the nucleotide sequences of our isolates to reference strains. The analysis revealed that while there is a high degree of nucleotide identity in segment A between most of the strains we evaluated, a particularly high degree of similarity was detected between our eight IBDV field isolates and reference vvIBDV strains (~95–99% similarity) (Figure 2A). Of note, vvIBDV reference strain UK661 shows >96% nucleotide similarity with all eight field isolates in segment A, with some strains like EDE14 showing >98% similarity. It is also worth noting the only reference strain we evaluated for Serotype II IBDV had a relatively low similarity to all other assessed strains.

#### 3.2.2. Segment B Nucleotide Analysis

Comparison of the nucleotide sequence for segment B reveals that the genome sizes of segment B in EDE14, NKL14, RPM14, and THI14 isolates are 2827 nts in length and have a coding region of 2649 nts (bases 112–2751). Details regarding the length, coding regions, and amino acids for all the isolates are shown in Table 1. Sequencing results from our field isolates revealed a high degree of sequence similarity for segment B among all isolates, with a nucleotide similarity of 95–99.7% for all strains (Figure 2B). Unlike segment A, the reference vvIBDV strains showed relatively low similarity to our field isolates, with most reference vvIBDV strains showing 89–92% similarity to the field isolates. On the other hand, reference strains from variant, classical, and attenuated IBDV showed a higher degree of similarity to the field isolates (~95–100%). Interestingly, field isolate BGE14 showed 100% sequence similarity to reference variant strain D78 for segment B. Of all eight field isolates, MDI14 appears to have the most sequence diversity, as the similarity of that strain with variant, classical, and attenuated IBDV reference strains is slightly lower than all other strains.

### 3.3. Segment A Subregion Analysis

#### 3.3.1. VP5 Capsid Protein

In the VP5 capsid protein, six major amino acid substitutions at positions R45G, F/L74I, H118N, P/S125H/N, W133R, and H134N are observed in various IBDV isolates as compared with known vvIBDV strains from Genbank (UK661, UPM, ZJ2000, and 88180). The complete aa substitutions in the VP5 capsid protein are provided in the Supplementary File for this article.

#### 3.3.2. Polyprotein (VP2–VP4–VP3)

- Viral Protein 2 (VP2)

**Figure 3:**
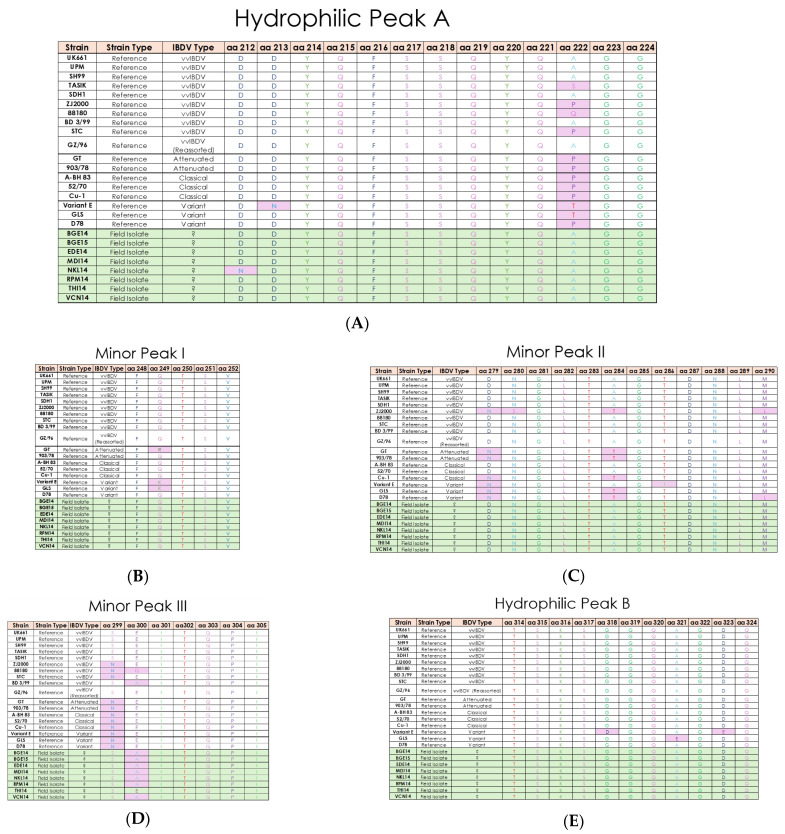

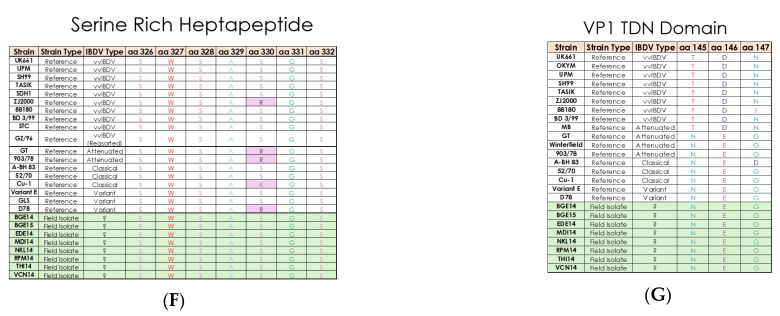
Multiple sequence alignment of field and reference IBDV strain amino acid sequences for (**A**) hydrophilic peak A; (**B**) minor peak I; (**C**) minor peak II; (**D**) minor peak III; (**E**) hydrophilic peak B; (**F**) serine rich heptapeptide; (**G**) VP1 TDN domain. Field isolates are shown in the green section, while reference strains are in white. Any amino acids showing variation compared to vvIBDV reference strain UK661 are highlighted in pink, except in panel (**G**).

Multiple sequence alignment of IBDV field isolates revealed that the VP2 hypervariable region is located from residues 206 to 350. The VPX protein (aa 1–512) revealed several amino acid substitutions and potential antigenic regions. Analysis of the VP2 sequence in all eight isolates of IBDV showed that there are four amino acid residues unique to most vvIBDV strains, which include the canonical vvIBDV residues A222, I242, I256, and S299. It was also observed that isoleucine is substituted by valine at residue 294 (I294V) in all eight isolates. Among these five amino acid residues, only A222 was located at the hydrophilic region of the VP2 variable region. In the present study, five hydrophilic regions are observed in all the isolates, which are hydrophilic peak A (aa 212–224), minor peak I (aa 248–252), minor peak II (aa 279–290), minor peak III (aa 299–305) and hydrophilic peak B (aa 314–324) (Figure 3). Analysis of hydrophilic peak A revealed that this region is conserved for all the IBDV isolates except in the NKL14 strain at 212 (D212N) (Figure 3A). Minor peak I and II and hydrophilic peak B were also conserved in all the IBDV isolates (Figure 3B,C,E). Minor peak III revealed one amino acid substitution at residue 300 (E300A) (Figure 3D).

The results of the present study revealed that aa A441 and F442 in the precapsid VP2 maturation site, aa A512 and A513 in the VP2-VP4 cleavage site, aa A755 and A756 in the VP4–VP3 cleavage site, and aa 756–760 in the C-terminus of VP3 are conserved in all the eight isolates. Apart from this, the VP2 protein reveals additional unique amino acid substitutions at 249(Q), 253(Q), A270T, 279(D), 284(T), L451I, and L481C in comparison with vvIBDV reference strains UK661, OKYM, and GZ2000, which are unique to Indian IBDV isolates. The complete amino acid substitutions at the VP2 polyprotein are given in the Supplementary File.

The serine-rich heptapeptide positions from aa 326 to 332 (SWSASGS) are conserved in the all the IBDV isolates and further indicate that these isolates belong to vvIBDV subtypes (Figure 3F). The change in aa at position L451I is observed in NKL14 and VCN14, which may affect pep46 peptide release during the VP2 maturation process.

- Viral protein 3 (VP3)

Analysis of the VP3 protein reveals two unique aa substitutions at G849E and V951I in all eight isolates of IBDV. The amino acid substitutions at V990I and 1005 (A) are observed in all the IBDV isolates except EDE14 and RPM14, where the substitutions are V990G and A1005G. The last five acidic residues at the C-terminus of VP3 (aa 756–760, which interact with the precapsid of VP2) are conserved in all the eight isolates. The sixteen amino acids of the VP3 C-terminus (aa 756–771, which interact with VP1) are also conserved in all the isolates. Several other amino acid substitutions are also revealed in the VP3 protein and are given in the Supplementary File. Changes are also observed in the putative RNA fixation site at aa V990A in BGE14, BGE15, MDI14, NKL14, THI14, and VCN14 isolates, and V990G is observed in EDE14 and RPM14 isolates. A single change at A1005G was observed in EDE14 and RPM14 isolates in the VP1 interaction site.

- Viral protease 4 (VP4)

All IBDV isolates have a substitution of I at aa 541 in Motif I, except BGE14 and VCN14, where it is substituted by C. Similarly, at amino acid 680, Y is observed in BGE14, BGE15, EDE14, MDI14, THI14, and RPM14, whereas C and S are observed in NKL14 and VCN14, respectively. It is observed that in all the isolates, the amino acid V is observed at 686, except in THI14 where it is substituted with G. The aa residue at 715 (typically S) is substituted with P in NKL14 and VCN14. The aa residue at 751 (typically D) is substituted with H in NKL14, RPM14, and VCN14. The amino acid substitutions in the VP4 protein of our isolates are given in the Supplementary File. No unique substitutions are found within the domain I and II (aa 576–584), Motif III (aa 644–661), substrate binding domain IV (aa 697–705), the catalytic triad (H546, D589, and S652), or the catalytic dyad (S652 and K692) in any of the eight isolates. The mutations I541V in BGE14, NKL14, and VCN14 and D751H in NKL14, RPM14, and VCN14 are reported as close to the cleavage site of VP4-VP3 and precursor VP2–VP4 junctions, respectively.

### 3.4. Segment B Subregion Analysis

Viral Protein 1

We first deduced the amino acid sequences of the VP1 protein (encoded by genome segment B) in the IBDV field isolates. Sequencing analysis reveals that segment B encodes an 880 amino acid VP1 protein. Details of the length, coding regions, and amino acids of all the isolates are shown in Table 1.

Multiple sequence alignment of the sequences of the VP1 protein in our IBDV field isolates and reference strains shows several unique amino acid substitutions. The VP1 has three domains in which five unique aa substitutions—namely V4I, K13T, I61V, D146E, and N147G—are found in the N-terminus (aa 1–167). These substitutions are believed to be involved in protein priming due to the presence of a putative guanylation site. The central domain is known to contain 5 RNA polymerase motifs (aa 168–658) and was determined to bear seven unique aa substitutions—namely E242D, A287T, C330W, C331L, K337I, Q341P, and S646G. As these residues are known for their polymerase activity, these substitutions may disrupt such activity. The C-terminus domain (aa 659–878) has two unique aa substitutions—namely P687S and P695K. These aa substitutions are conserved in all the eight IBDV isolates. The other amino acid substitutions of VP1 protein are given in the Supplementary File.

The presence of motif TDN at aa positions 145–147 is considered as a putative aa marker for vvIBDV across the VP1 protein. In the present study, the motif TDN is substituted by the motif NEG in all the eight isolates (T145N, D146E, and N147G) (Figure 3G). Changes in the catalytic domain (aa 390–393) of VP1, M390L, and D393E are also observed in BGE14, EDE14, NKL14, RPM14, and VCN14 isolates. The putative RNA-dependent RNA polymerase (RdRp) region (aa 416–422) and RdRp motif (aa 528–541) in the VP1 protein are conserved in all the IBDV isolates.

### 3.5. Comparison of Both 5′ and 3′ Ends of Segment A and B

Comparison of the 5′ terminal sequences of both genome segments (A and B) reveals that the 32-nucleotide consensus sequence is highly conserved in both the segments of all the isolates, except in BGE15, where variation is observed in segment B. Analysis of the 3′ terminal sequences of both segments (A and B) of eight IBDV isolates and serotype II reveals the presence of highly conserved pentamer –GCGGU.

Perfect inverted terminal repeats (ITRs) are present in the highly conserved segment-specific regions in both genome segments. In segment A, an ITR of six nucleotides is localized at nucleotide positions 36 to 41 and 3255 to 3260. In segment B, two ITRs are found: ITR segment B1, consisting of nucleotides 1 to 7 and 2796 to 2802, and ITR segment B2, formed by 12 nts, located at positions 87 to 97 and 2768 to 2778.

### 3.6. Putative Binding Sites for 18S rRNA in the 5′ NCR

The nucleotide sequence CUCCUC is highly conserved in segment A (positions 71–76) as well as in segment B (positions 86–91). This motif is flanked at its 5′ end by the nucleotides UGG, either directly, as in segment B, or with two intervening nucleotides, as in segment A. The 3′ end is flanked by UUCU, again, directly in segment B and with one intervening nucleotide in segment A. BLAST analysis (https://blast.ncbi.nlm.nih.gov/Blast.cgi accessed on 15 March 2024) revealed that these regions are partially complementary to the 3′ end of chicken reticulocyte 18S rRNA. Furthermore, the CUCCUC motif is part of the ITR segment B2. Interestingly, in both segments, respective start codons for the nearest ORF (VP5 gene on segment A and VP1 gene in segment B) are located exactly 19 nts downstream from this putative rRNA binding site.

### 3.7. Antigenicity Index

**Figure 4:**
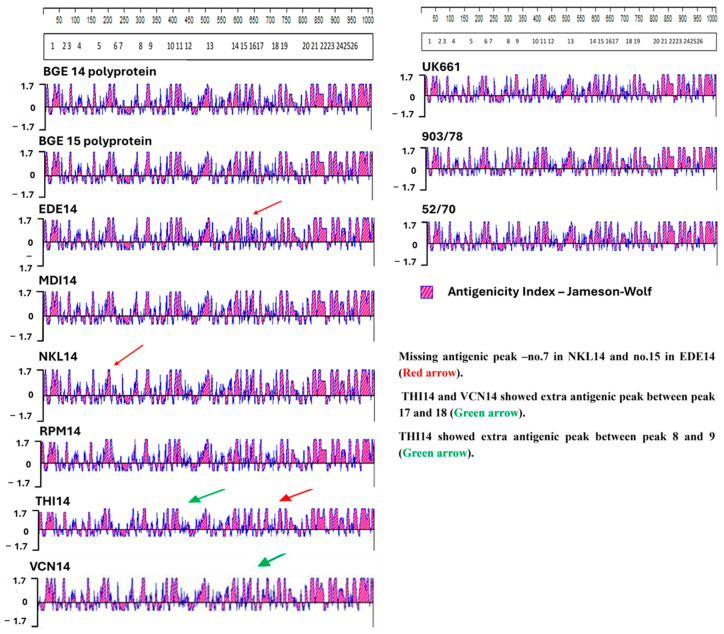
Antigenicity index analysis. The scales at the top represent the positional coordinates of amino acids along the VP2 gene. The boxed numbers below show the positions of known antigenic peaks in the VP2 gene. The antigenicity index of reference strains UK661, 903/78, and 52/70 are shown on the right. Field isolate strains are shown on the left. Red arrows indicate missing antigenic peaks compared to reference strains, while green arrows indicate extra antigenic peaks compared to the reference strains.

Antigenicity index analysis predicts regions of a viral protein likely to elicit an immune response, helping identify key epitopes. In IBDV, it typically reveals conserved antigenic peaks in the VP2 hypervariable region; differences in this pattern can indicate strain-specific immune recognition. In the present study, classical strain 52/70 shows an extra antigenic peak between numbers 7 and 8 and between 8 and 9 compared with both field IBDV isolates of the present study and UK661. Comparison of our eight Indian IBDV isolates with UK661 reveals that the field isolates demonstrate missing antigenic sites at number 7 in NKL14 and number 15 in EDE14 (Figure 4). The isolates THI14 and VCN14 show an extra antigenic peak at numbers 17 and 18. The isolate THI14 also reveals extra antigenic peaks at numbers 8 and 9 compared with the UK661 isolate. Other isolates such as BGE14, BGE15, MDI14, and RPM14 exactly match with the antigenic peak of vvIBDV isolate UK661.

### 3.8. Phylogenetic Analysis

**Figure 5:**
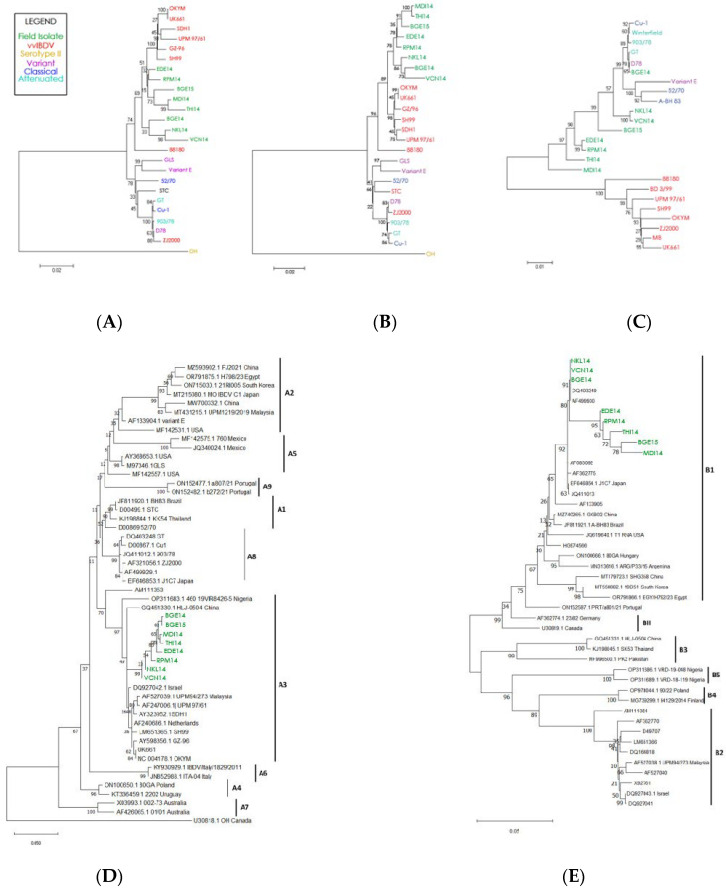
Pathotype-based phylogenetic analysis of: (**A**) complete genome segment A; (**B**) the hypervariable (HVR) region of the VP2 protein (located within segment A); (**C**) complete genome segment B. Strain names are color-coded based on IBDV strain type. Genotype-based phenotypic analysis of (**D**) complete genome segment A; (**E**) complete genome segment B.

Phylogenetic analysis of the complete genome of the segment A nucleotide and polyprotein amino acid sequence resulted in a distinct cluster formed by our eight IBDV field isolates, indicating that the sequence of segment A is highly conserved across field isolates (Figure 5A). The group of field isolates also clustered closely to the vvIBDV reference strains, suggesting a high degree of sequence similarity between field isolates and the reference vvIBDV. Phylogenetic analysis based on the VP2 hypervariable region nucleotide and amino acid sequences also reveal that all the isolates of this study are grouped into a separate cluster along with already reported very virulent European, Asian, and Japanese isolates, indicating their close evolutionary relationship (Figure 5B). These results clearly indicate that segment A of all the isolates belongs to vvIBDV.

Phylogenetic analysis of the complete nucleotide sequence of segment B reveals that all the isolates of the present study are grouped in a cluster containing classical, attenuated, and variant IBDV lineage (Figure 5C). For segment B, the reference vvIBDV strains clearly cluster independently of the field isolates. These results clearly indicate that segment B of the IBDV isolates in the present study belongs to either the classical virulent or classical attenuated strain.

#### 3.8.1. Phylogenetic Analysis of Segment A

Phylogenetic reconstruction of segment A using the updated unified classification scheme (A0–A9) grouped all eight Indian field isolates (BGE14, BGE15, MDI14, THI14, EDE14, RPM14, VCN14, and NKL14) within genogroup A3, corresponding to the very virulent (vvIBDV) lineage (Figure 5D). The isolates formed a strongly supported monophyletic cluster alongside established vvIBDV reference strains such as UK661 and OKYM, with bootstrap values exceeding 90%, confirming their close genetic affinity to globally circulating vvIBDV strains. None of the isolates associated with classical (A1), variant (A2), attenuated (A8), or geographically distinct A4–A7 groups, indicating a consistent A3-type circulation across the sampled outbreaks. Their close relationship to contemporary Asian vvIBDV strains from China, Southeast Asia, and the Middle East further suggests that the prevailing Indian strains belong to the wider, internationally distributed A3 lineage.

#### 3.8.2. Phylogenetic Analysis of Segment B

Phylogenetic analysis of segment B using the updated genogrouping system (B1–B5) revealed that all eight Indian isolates (BGE14, BGE15, MDI14, THI14, EDE14, RPM14, VCN14, and NKL14) clustered exclusively within the B1 lineage, with strong bootstrap support (63–95%) (Figure 5E). Within B1, the isolates segregated into two well-defined sub-clusters, one comprising NKL14, VCN14, BGE14, BGE15, DQ403249, and AF499930, and the other consisting of THI14, MDI14, RPM14, and EDE14, indicating that despite geographic and outbreak-level variation, all viruses share a common classical-like or attenuated-like segment B backbone. None of the isolates grouped with B2 (vv-like), B3 (early Australian-like), B4 (Polish/Tanzanian/Finnish-like), or B5 (novel Nigerian-like) reference lineages, and the use of representative strains from each genogroup further confirmed their exclusive placement in B1. Genotypically, all isolates exhibited conserved B1-defining signatures, including the NEG motif at residues 145–147 and the hallmark D242 residue, both absent in vvIBDV-associated B2 strains, thereby reinforcing their classification as classical/attenuated B1-type segment B viruses.

### 3.9. Detection and Analysis of Recombination Events

**Figure 6:**
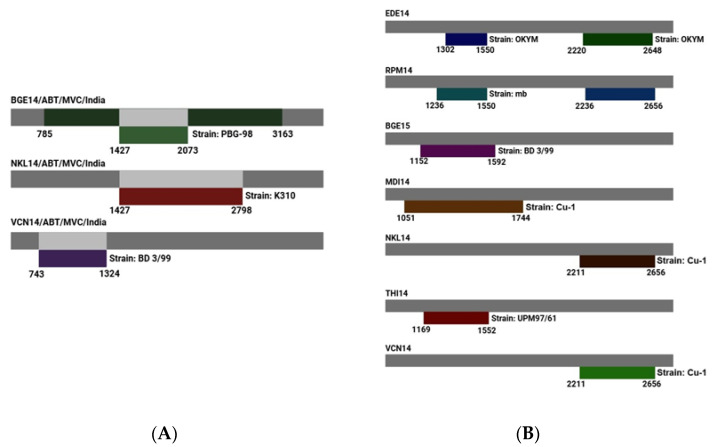
Predicted recombination events detected for (**A**) genome segment A; (**B**) genome segment B. Field isolate strains are shown as gray bars, with suspected reference donor strain shown below them. Amino acid positions for the predicted recombination events are noted below each set of bars.

The detection and analysis of recombination events in IBDV involve identifying genetic exchanges between different viral strains, which contribute to viral evolution and diversity. In segment A, all six algorithms utilized predicted four recombination events in many IBDV isolates. Segment A of BGE14 is predicted to have two recombination events, which are localized in the coding region of 1427–2073 nts and 785–3163 nts, respectively. Segment A of NKL14 and VCN14 shows only one recombination event (1427–2798 nts for NKL14 and 743–1324 nts in VCN14) (Figure 6A). The two recombinant events for BGE14 were detected by the RDP4 program (1427–2073 nts and 785–3163 nts), with reference strains PBG-98 as a minor parent and BD-3199 and T09 very virulent field isolates from Bangladesh and Nigeria as predicted major parents. For NKL14, the major recombinant parent is BD-3/99 and minor recombinant parent is K310, whereas for VCN14, K310 is the major recombinant parent and BD-3/99 is the minor recombinant parent.

In segment B, all six algorithms utilized predicted nine recombinant events. Segment B of EDE14 and RPM14 is predicted to have two recombination events, which are localized at 1302–1550 nts and 2220–2648 nts for EDE14 and 1236–1550 nts and 2236–2656 nts for RPM14. Segment B of BGE15, MDI14, NKL14, THI14, and VCN14 shows one recombination event (Table 3). Two recombination events were identified in EDE14, involving segment B of the Cu-1 classical virulent isolate as the major parent and the OKYM strain as the minor parent. Similarly, RPM14 exhibited two recombination events, with segment B of Cu-1 and A-BH 83 classical virulent isolates serving as major parents and OKYM along with the attenuated MB strain acting as minor parents. There is only one recombination event predicted for BGE15, MDI14, NKL14, VCN14, and THI14 of segment B of IBDV. All these isolates have the Cu-1 segment B of classical virulent isolate as the major parent, whereas BGE15, VCN14, NKL14, and MDI14 have BD 3/99, OKYM, MB, and UPM G7/61 as the minor parent, respectively.

Interestingly, for segment B in all the isolates (except one recombination event in RPM14), the Cu-1 classical virulent isolate is regarded as a potential parent for recombination events that are localized in the coding region of RdRp, even though their break point positions start and end at different nucleotides. This result clearly suggests that segment B of all the isolates of the present study might have been obtained from isolate Cu-1 (classical virulent) by reassortment (Figure 6B).

## 4. Discussion

One of the major problems observed in the poultry flocks of India is the frequent outbreaks of IBD despite the extensive use of available IBD vaccines. This failure in vaccination could be due to the genetic drift or mutations observed in VP2 (which is a major protective antigen of IBDV), which may result from immunological pressure or genetic reassortment between more than one strain [43,44]. The polygenic nature of IBDV and the role of segments A and B in the pathogenesis of vvIBDV, described by several researchers [4,45,46], have clearly indicated that the characterization of IBDV strains based on both genome segments is essential to understand the epidemiological behavior of this viral agent.

Genome reassortment and recombination mutations have been speculated to occur in IBDV and contribute to the emergence of new strains. In the recent past, evidence regarding natural reassortant viruses have been reported in India, Pakistan, China, Europe, and Zambia [47,48,49,50,51,52]. This study emphasizes the genetic structure of both IBDV genome segments and explores the emergence and spread of vvIBDV and novel reassortants through complete genome sequencing, amino acid comparison, and phylogenetic analysis.

### 4.1. Complete Genome Sequence Analysis of Segments A and B

#### 4.1.1. Analysis of Segment A Genome

VP2 is the major capsid protein in the IBDV particle and contains antigenic determinants encoded by seven amino acid residues (A222, I242, Q249, S251, I256, I294, and S299), which are used as markers for vvIBDV; isolates which carry at least three of seven aa residues are categorized as the very virulent strains. All these amino acid residues are located in the VP2 hypervariable regions, which have a high tendency for nucleotide exchanges [53]. In the present study, analysis of the VP2 hypervariable region revealed that all eight isolates carried these five amino acids, A222, I242, Q249, I256, and S299, highly conserved for the vvIBDV strains [23,24,54]. However, all the isolates also carried a substitution of isoleucine by valine at residue 294 (I294V). Position 294 is located within the minor hydrophilic loop of VP2, and although the I→V change is a conservative hydrophobic substitution, its consistent presence across multiple outbreaks suggests a region-specific evolutionary pattern, potentially marking V294 as an emerging feature of South Asian vvIBDV-like strains. This substitution may subtly alter VP2 loop conformation, with possible effects on receptor interaction, antigenicity, and vaccine matching. However, targeted reverse-genetics and structural modelling studies will be required to confirm the functional and antigenic impact of I294V.These results clearly indicate that the VP2 hypervariable region can be used as the target for presumptive molecular or antigenic identification of putative vvIBDV. However, Boot et al. [32] and Escaffre et al. [55] observed that amino acid residues N279 and T284 were highly conserved in classical attenuated strains as compared to residues D279 and A284 in the VP2 hypervariable region of vvIBDV strains. Although residues I242, I256, V294, and S299 are not directly in hydrophilic regions, their locations are much closer, and they are unique residues that have been used as indicators of the very virulent subtypes of IBDV. Isolates of IBDV that carry at least three of the seven aa residues are categorized as vvIBDV subtypes. The isolates from the present study contain four of these unique residues in their hydrophilic regions and are therefore considered to be vvIBDV strains.

Comparison of the eight IBDV isolates of the present study with the attenuated IBDV strains revealed that among the 10 aa residues at positions 222, 242, 253, 256, 279, 284, 290, 299, 330, and 450 of the attenuated strains, the isolates also showed the same aa at positions Q253, D279, and A284, indicating that all the isolates of the present study were vvIBDV. The findings were similar to those of Brandt et al. [28], Boot et al. [56], and Van Loon et al. [29], who opined that attenuated viruses had changes in aa positions at 253, 279, 284, 290, and 330, further evidence that all the isolates of the present study are vvIBDV. However, based on the previous reports, it was evident that even though these three amino acids could be used for the differentiation of attenuated IBDV and vvIBDV, these three amino acids were found to be highly conserved in both vvIBDV and cvIBDV strains and hence cannot be used for differentiation of these strains [28,29].

The isolates NKL14 and VCN14, despite being vvIBDV, have amino acid substitution L451I within the VP2 processing site. This change is in contrary to the findings of Da Costa et al. [57], who opined that substitutions or deletions within the VP2 processing site (442–512) might be lethal for the virus, resulting in decreased virulence. In this study, all the eight isolates have Q253, as indicated by Mundt [26], who opined that the presence of Q253 in IBDV significantly affects its virulence and cell tropism. It is further observed that the last predicted antigenic region is within the VP2 carboxyl terminal and is conserved among the sequenced isolates. Similarly, it has been found that in the VP2 carboxyl domain, aa 50–60 may play a role in VP2–VP2 and VP2–VP3 interaction and capsid formation [58,59]. In all the eight IBDV isolates, the monocistronic ORF2 encoding precursor VPX–VP4–VP3 polyprotein cleaved at amino acids (aa) A512-A513 for VPX–VP4, indicating that this region is highly conserved [60,61]. It is also evident that in the VP2 antibody binding region, aa 206–350 is conserved in all the isolates except NKL14 (D212N in major hydrophilic peak A) and THI14 (E300A in minor peak III). The variations in these aa positions could affect the mechanism responsible for inducing host-protective neutralizing antibodies [62,63]. Similarly, Yamaguchi et al. [64] opined that amino acid substitutions between the two major hydrophilic A and B regions I240F and I294V in minor peak II may significantly affect the virulence of the isolates.

Analysis of the serine-rich heptapeptide region of all the eight isolates of IBDV revealed that the region is highly conserved and no aa substitutions are revealed in the serine-rich motif SWSASGS (aa 326–332). The results of our study are supported by the findings of Eterradossi et al. [23], who observed that the attenuated and vaccine strains have a serine-rich motif in SWSARGS (aa 326–332), indicating that all the isolates in this study belong to vvIBDV [65]. Comparison of the deduced amino acid profiles of all the eight Indian IBDV isolates of the VP2 protein vvIBDV strains (UK661, OKYM, and GZ2000) revealed that all eight of our Indian isolates have four unique amino acid substitutions at A270T, 284(T), L451I, and L481C. These variations might lead to antigenic variants with a different pathogenesis and cell tropism.

Anti-VP4 monoclonal antibodies have been used to discriminate a pathogenic infection (vvIBDV) from non-pathogenic infection (attenuated strain). Hence, variation in the VP4 protease might play a role in viral adaptation and pathogenicity [66]. The VP4 protease of the vvIBDV contains four diagnostic residues: Y680, G681, S715, and D751. Among the four amino acid residues, G681 was conserved in all the isolates. Amino acid at position Y680 was conserved in all the isolates except NKL14 (Y680C) and VCN14 (Y680S), S715 was conserved in all the isolates except NKL14 and VCN14 (S715P), and D751 was conserved in all the isolates except NKL14, RPM14, and VCN14 (D751H). The results of this study clearly indicate that mutations or substitution in the VP4 protease may affect the polyprotein cleavage and may significantly affect pathogenesis [67]. The substitution of I541V in the VP4 protein in isolates BGE14, NKL14, and VCN14 is within Motif I. No substitutions were found in any of the isolates between serine-protease Motifs I and II.

The amino acid substitutions at flanking sequences of VP4–VP3 cleavage sites have been reported to influence the efficiency of processing and assembly of the virus particles [26]. In the present study, there was only one unique amino acid substitution (I541V) in isolates BGE14, NKL14, and VCN14, which are close to the VP2–VP4 cleavage site and might affect virulence through the processing of the polyprotein by the viral cleavage protease. In addition, the other amino acid changes unique to all the eight isolates in VP3 and VP4 may also be involved in the increased virulence. It has been shown that exchange of the C-terminus of VP3 from vvIBDV resulted in attenuation of the virus. The amino acids from positions 997–1012 in the C-terminus of VP3 are known to be essential for interaction of VP3 and VP1 and can influence virus replication and capsid formation [68]. The substitution of A1005G in vvIBDVs, as compared to non-vvIBDVs, could contribute to differences in VP1 binding activity and virus replication and hence contribute to the phenotypic differences between very virulent and non-very virulent classical serotype I strains.

Analysis of the VP3 protein reveals two unique aa substitutions (G849E and V951I) in all eight field isolates of IBDV; substitution Q993T is observed in EDE14 and RPM14 while Q993L is observed in THI14 within the double strand (ds) RNA-binding domains of the VP3 region. It is observed that the aa residues at positions Q981 and A1005 are conserved in all the Indian IBDV isolates except EDE14 and RPM14 (A1005G) in the VP1-binding domain, similar to the findings of Chong et al. [67] and Tacken et al. [69], who opined that residue A1005 is characteristic of vvIBDV in VP3, significantly affecting pathogenesis and playing an important role in IBDV replication and genome packaging.

All the vvIBDV isolates in the present study had unique mutations at position 990 (V990A) except in RPM14 and EDE14 (V990G) and at position 1005 (A1005G), only in RPM14 and EDE14. In the present study, changes are observed in the aa position 990 in the VP1–VP3 interactive domain, which could be attributed to the fact that VP1 is known to interact with VP3 and VP3 interacts with the genome segments, which could have modified aa at polyprotein positions between 981 and 990 [70,71].

The P5 cytotoxic protein has been implicated as an important factor for viral release, the induction of apoptosis, and pore formation in the cells [72,73]. Although previous analysis of VP5 did not reveal amino acids unique to the very virulent pathotype, amino acid substitutions could affect VP5 function [74]. In the present study, most of the IBDV isolates have five major amino acid substitutions in the VP5 capsid protein at positions R45G, F/L74I, H118N, P/S125H/N, W133R, and H134N compared to reference very virulent IBD strains (UK661, UPM, ZJ2000, and 88180). Among all the eight isolates, VCN14 harbors the most aa mutations, with 17 aa substitutions being detected, followed by BGE15 with 6 aa substitutions. The strains EDE14, MDI14, NKL14, and THI14 have five aa substitutions, whereas RPM14 and BGE14 have four and three aa substitutions, respectively. According to Yao and Vakharia [75], it could be speculated that mutations in the VP5 region might not exert a significant impact on the pathogenesis of the isolates. Instead, it appears that the pathogenicity of the isolates is upheld by the intact segment B, which shares genetic relatedness with VP5 [31,32,33].

Inspection of the VP5 sequence revealed the presence of a polycationic C-terminal region spanning aa residues 132–143 and containing three closely spaced clusters formed by two or three consecutive basic amino acid residues, namely K132 and R133, in all the isolates except in EDE14 and RPM14, where it was K132 and W133. It was also observed that the amino acid residues at K136, R138, R142, and K143 were highly conserved in all eight isolates. Similarly, Hernández et al. [76] stated that the virulence level of IBDV depends on the existence of one conserved amino acid substitution at R133W and observed that amino acid residues 132–143 of VP5 could be used for differentiating classical virulent from very virulent serotype I strains because of its conserved nature.

The stem-loop structure of the 3′ NCR of segment A is an important functional determinant [77] and plays a role in protein-primed RNA synthesis [78] and RNA packaging [30]. The inverted terminal repeat (3255–3260 nts) at the 3′ NCR of segment A is well conserved in all the isolates, and the 18S rRNA binding domain (nts position 71–76) within the 5′ NCR of segment A is also conserved. Any mutational changes in these regions may affect the transcription and translation efficiency [79].

The nucleotide sequence CUCCUC is highly conserved in segment A (residues 71–76) as well as in segment B (residues 86–91). This motif is flanked at its 5′ and 3′ ends by conserved nucleotides, either directly as in segment B or with two intervening nucleotides as in segment A. BLAST analysis revealed that this region is partially complementary to the 3′ end of the chicken reticulocyte 18S rRNA [80]. However, no clear evidence is available to correlate the presence of the conserved sequence motif CUCCUC as a binding site for 18S rRNA and its influences on translation and/or replication. Similar observations have been made by Deng and Brock [81] in Pestiviruses and Le et al. [82] in Picornaviruses. Nucleotide sequences at the termini of viral RNA specify signals for RNA replication, transcription, and translation [83].

The conserved terminal sequences in the IBDV genome segments may represent virus-specific signals, whereas the segment-specific ITRs and/or segment-specific 3′ terminal sequences might function as segment recognition signals, although the significance of segment-specific sequences of inverted complementarily in viral RNAs is not clear [84].

#### 4.1.2. Analysis of Segment B Genome

The RNA-dependent RNA polymerase (RdRp) has been implicated in determining virulence in several viruses, including the measles virus [85], rinderpest virus [86], human parainfluenza virus type 2 [87], and influenza viruses [88]. In the case of IBDV, the RdRp activity is carried out by the VP1 protein, which facilitates viral genome replication and RNA transcription. VP1 is known to associate with both termini of the dsRNA genome and with the inner capsid surface. Its interaction with VP3, forming VP1–VP3 complexes, is essential for efficient viral replication and the proper assembly of IBDV particles. Therefore, modifications in VP1 may influence virulence by altering polymerase activity or by disrupting virus particle morphogenesis. The direct role of VP1 in replication and pathogenicity has been experimentally demonstrated by Liu and Vakharia [89].

In the present study, it was observed that segment B was highly conserved among all the isolates. The VP1 protein in MDI14 has three more amino acids and EDE14, NKL14, VCN14, RPM14, and THI14 have one more amino acid as compared to BGE14 and BGE15. Similar variations in the amino acids of VP1 in IBDV had been previously described by Islam et al. [33]; however, no reports are available to demonstrate the significance of the variation in different strains. It was also observed in all the isolates of the present study that the amino acid position at 147 is substituted (N147G). Similar variations have been previously described by Brandt et al. [28] for classical and variant strains (Cu-1, GT, Winterfield, variant E, and D78). Several researchers have suggested that this substitution may play an important role in cell-specific replication and virulence [90]. In the present study, it was observed that the phosphorylation, glycosylation, NTP-binding motifs, and RNA-dependent RNA-polymerase (RdRp) motifs (aa 528–541) are highly conserved between all strains analyzed. The results were in agreement with the findings of Duncan et al. [91], Gorbalenya and Koonin [92], Gorbalenya et al. [93], and Shwed et al. [68].

Although segment B is highly conserved among the field isolates, a few exceptions are identified. The crystal structure of the VP1 protein reveals three domains, namely the N-terminus (aa 1–167), central polymerase (aa 168–658), and C-terminus (aa 659–878). In the N-terminus domain, seven unique amino acid variations are observed at positions V4I, K13T, I61V, T145N, D146E, and N147G. These amino acids substitutions are similar to that of classical and variant strains of IBDV, and this variation may affect the protein priming as it possess the putative guanylation site at the N-terminus of VP1 [94]. In the central polymerase domains, 11 amino acid substitutions are observed at positions E242D, A287T, C330W, C331L, K337I, Q341P, S343P, M390L, D393E, P546L, and P562S. These amino acid variations may affect viral replication, as the central polymerase domain is known to be involved in this process [95]. In the C-terminus domain of the VP1 protein, two unique amino acid substitutions at positions P687S and P695K were detected.

The triplet amino acids (TDN) in positions 145/146/147 and residue E242 in VP1 have been linked to vvIBDV [96,97]. Therefore, comparisons of amino acid replacements based on this pattern among all Indian IBDV sequences revealed that the pattern of the triplet amino acids is NEG (145–147) and the amino acid at position 242 was D in all the isolates, indicating that segment B of the isolates in the present study is similar to those of non-vvIBDV strains and vaccine strains. All the Indian field isolates showed the signature D242 present in non-vvIBDV strains, suggesting a possible attenuation [98].

It has been documented that the VP1 protein of vvIBDV isolates has 15 characteristic amino acid residues (V4, K13, I61, T145, D146, N147, E242, A287, M390, D393, K508, S511, P562, P687, and R695) [99]. Analysis of the study isolates revealed that out of these 15 residues, many are substituted, including V4I, K13T, I61V, T145N, D146E, N147G, E242D, A287T, M390L (except in BGE15, MDI14, and THI14), D393E (except in BGE15, MDI14, and THI14), K508R (except in BGE15, EDE14, MDI14, RPM14, and THI14), S511R (except in BGE15 and MDI14), P562S (except in MDI14), P687S, and R695K. All eight isolates of this study differed from vvIBDV in each of the 15 characteristic amino acid residues in segment B, which make it identical to most of the classical, variant, attenuated and reassortant IBDV strains. Among these 15 unique amino acids, 8 aa residues (146, 147, 242, 287, 390, 393, 562, 687, and 695) are located in the external regions of the proposed RdRp motifs and GTP binding sites [68]. These data will be useful for the analysis of the epidemiology and evolutionary characteristics of IBDV in India.

The N-terminal domain of IBDV VP1 folds into a mixed α/β structure; this domain has previously been associated with the protein priming process and is known to interact with the fingers and thumb domains of VP1 [95]. The F domain is associated with nucleotide recognition and binding, and it also plays a role in the interaction with the RNA template during replication. Other regions, including domain D beginning at residue 252, which contributes to maintaining structural stability and the C-terminal domain, exhibit strong conservation not only across various IBDV strains but also throughout the entire Birnaviridae family [100]. Analyses of the genetic variation among Indian IBDV isolates have identified a novel natural reassortant, characterized by segment A originating from vvIBDV strains and segment B derived from non-vvIBDV strains within the circulating population.

All the isolates have conserved amino acid residues at V61, N145, G147, and D242 in VP1 (segment B), which is similar to that of classical strains, while amino acid residues of the VP2-HVR region (segment A) are the same as those of very virulent strains. Furthermore, all the eight isolates grouped based on segment A and the VP2 hypervariable region-based phylogenetic tree shows that they are very virulent, whereas the isolates formed a cluster with classical attenuated strains in the tree constructed based on the nucleotides of segment B. The phylogenetic analysis based on VP2-HVR and VP1 revealed genetic differences, which may be inferred as the evolutionary changes in segment B of all the eight Indian isolates and could be used for investigation of natural genome reassortment.

### 4.2. Genetic Reassortment Between Segments A and B of Indian IBDV Isolates

Analysis of the demographic history of segment B in non-vvIBDV lineages indicates a downward trend in genetic diversity. This reduction is likely associated with the widespread introduction of effective vaccination programs targeting classical and low-virulence strains soon after the disease was first recognized. Since most conventional live IBDV vaccines are derived from classical virulent strains [100], repeated exposure of field viruses to vaccines with closely related genomes may have imposed recurrent population bottlenecks, ultimately reducing overall viral fitness through natural selection pressures [101]. Although newer vaccine platforms such as subunit formulations, recombinant live vaccines, and DNA-based vaccines were developed to enhance protection against vvIBDV, several challenges persist. These include the potential reversion of vaccine strains to virulence [97], the inadequate induction of robust immunity [102], and the inherently rapid evolutionary rate of IBDV, all of which complicate effective disease control through vaccination.

The expansion of vvIBDV strains was linked to a reassortment event of the genome (segment B) of IBDV with a mutant VP2 background, which caused a sudden increase in virulence of these kind of strains [43]. The vvIBDV strains have been kept antigenically and genetically homogeneous, spreading to most countries for at least two decades. However, the recent isolation of vvIBDV strains from India in the present study with rare natural segment B reassorted has evidenced a possible change in the genetic structure and stability of vvIBDV strains. Similarly, Ingrao et al. [103] suggested that it is probably only a matter of time until vvIBDVs are replaced by an emerging strain with new antigenic or pathotypic properties. In addition, He et al. [48] had reported that reassortant IBDV strains were dominantly prevalent in southern China during 2000–2012. Several studies have confirmed that reassorted IBDV and vvIBDV strains possess different biological characteristics [45]. According to reports by He et al. [48] and Qi et al. [104], vv-A/Uniq-B IBDVs (HLJ0504-like strains) have been identified as prevalent strains in China. More recently, these IBDVs have also been identified in Venezuela [45], Nigeria [105], India [4], and Algeria [106]. In Pakistan, unique segment-reassortant IBDVs (vv-A/Uniq-B), carrying segment A from vvIBDV and segment B from one unique ancestor, were identified in field outbreaks [46].

The results of the present study clearly indicate that in all the eight isolates, segment A is characteristic of vvIBDV while segment B is characteristic of non-vvIBDV, indicating a higher possibility of natural reassortment. The reason for the emergence of this natural reassortment may be attributed to the emergence of vvIBDV strains in India during 1992 [107]. Hence, the reassortment event in Indian isolates seems to have occurred between the introduction of the novel vvIBDV strains in India around 1992 and circulation of the attenuated IBDV strains among the Indian poultry population, starting in 1971 [108].

The phylogenetic analyses of our isolates revealed genomic reassortment between segments A and B of IBDV. Kasanga et al. [95] observed similar natural reassortment in IBDV strains with genome segment A from very virulent and segment B from classical attenuated IBDV strains in Africa.

The demonstration of such a strain could suggest the existence of previously unrecognized segment B lineages, which could be associated with a vvIBDV-like segment A and/or a possible reassortment in vvIBDV (segment A). Several researchers have reported natural reassortment in IBDV [21,109,110,111]. The reason behind natural reassortment could be because double-stranded RNA viruses have a high rate of mutation, recombination, and reassortment [112].

Phylogenetic and population analyses of vvIBDV indicate that its emergence can be traced to a reassortment event in which an endemic IBDV segment A combined with a segment B of previously unknown origin [43]. The subsequent global dissemination of vvIBDV suggests that selective pressures favored the stable co-evolution of these two genome segments, reinforcing this successful viral phenotype [33,45]. In fact, one study reported that nearly 70% of examined IBDV isolates showed evidence of coordinated evolution between segments A and B [45]. Despite this conserved co-evolutionary pattern, naturally occurring reassortant vvIBDV strains have been identified in areas where both vvIBDV and non-vvIBDV serotype 1 viruses circulate. These reassortants typically retain a vvIBDV-type segment A while acquiring segment B from a non-vvIBDV strain [45,113,114]. Additionally, two unusual natural reassortants detected in China displayed the opposite configuration: segment A originated from an attenuated vaccine strain, whereas segment B was derived from a vvIBDV strain [112,113,114].

Furthermore, detailed sequence and phylogenetic analyses based on the nucleotides of the complete genome of segment A and VP2-HVR revealed clustering of all the eight isolates (both partial and complete genome) with the very virulent genotype, whereas segment B (VP1 gene) formed a separate cluster related to classical, variant, and attenuated strains. The clustering pattern of the isolates in the phylogenetic tree was the same, with partial sequences from these segments. These findings indicate the possibility that VP2-HVR and the part of the VP1 corresponding to 332 amino acids of the N-terminus are the best representatives of the genome segments A and B of IBDV, respectively.

The updated phylogenetic analysis performed using the unified AB genotype classification strongly demonstrates that all eight Indian isolates cluster within genogroup A3, confirming their identity as very virulent (vvIBDV) strains. Their tight monophyletic grouping and conserved VP2 amino acid markers (A222, I256, I294, S299) highlight the genetic homogeneity of segment A and align with long-standing vvIBDV endemicity across India and Asia. By incorporating reference representatives for all A genogroups (A1–A9), as recommended by [35,36], the revised topology robustly supports this A3 classification and removes the ambiguity associated with earlier VP2-only phylogenies. Notably, unlike the recently expanding A2dB1b “novel variant” lineage reported in Asia, Africa, and South America [37], the current Indian isolates continue to retain a classical vvIBDV-type segment A.

Segment B analysis further revealed that all isolates belonged to genogroup B1, a lineage commonly associated with classical or attenuated strains rather than very virulent viruses. Their consistent B1-defining signatures—including the NEG motif at positions 145–147 and residue D242—clearly distinguish them from B2 vvIBDV-like segment B profiles and indicate that the circulating viruses likely arose from reassortment between vvIBDV-derived segment A (A3) and classical-like segment B (B1) [35]. Such A3B1 reassortants have been increasingly reported across Asia and are linked to altered virulence, vaccine escape, and broader evolutionary adaptability [36]. The concurrent circulation of vvIBDV-type segment A with classical-like segment B in Indian flocks underscores ongoing natural reassortment under field conditions and highlights the need for the continuous genomic monitoring and refinement of vaccination strategies.

However, in contrary to the findings of the present study, Wei et al. [112] isolated and characterized the genomic reassortant IBDV strain ZJ2000 from severe field outbreaks and observed that the natural genetic reassortment was, with segment A from attenuated and segment B from very virulent strains of IBDV, respectively. Considering the above facts, our data provide direct evidence of the genetic reassortment of IBDV in nature, which may play an important role in the evolution, virulence, and host range of IBDV. Our data also suggest that VP2 is not the sole determinant of IBDV virulence, and that the RdRp protein VP1 may play an important role in IBDV virulence.

**Figure 7:**
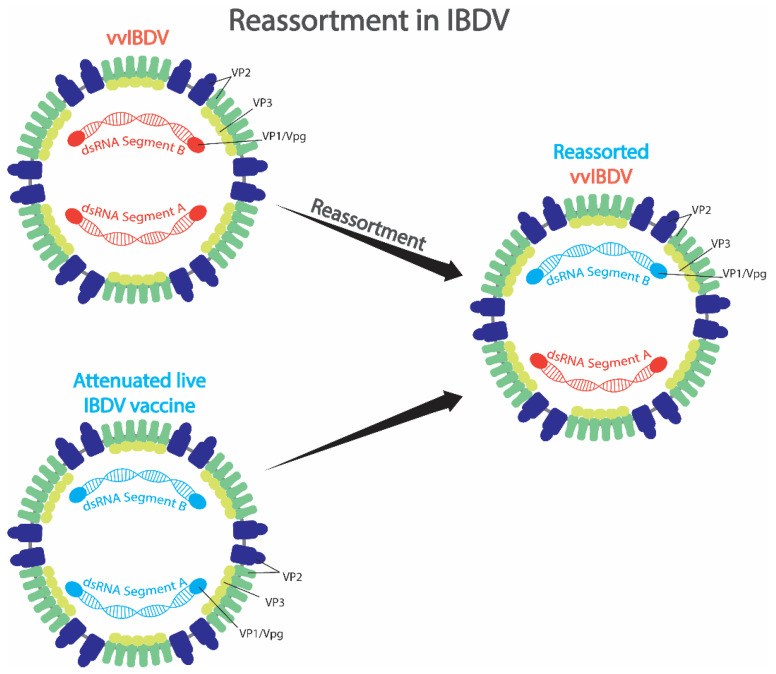
Model showing predicted reassortment events in IBDV, which led to the emergence of the field isolates evaluated here.

The potential pathways through which genomic reassortment occurs in IBDV under natural conditions are illustrated in Figure 7. Reports indicate that conventional live vaccines offer limited protection against vvIBDV [21,115]. Consequently, vvIBDV outbreaks in flocks previously vaccinated with live attenuated vaccines may result in co-infection of the same bird with both the field vvIBDV strain and the vaccine strain. When these viruses replicate simultaneously within the bursa, the primary target organ, there is an opportunity for exchange of their double-stranded RNA genome segments, giving rise to novel reassortant viruses. The detection of such reassortants in the field highlights an additional concern regarding live vaccine usage. Beyond the risk of reversion to virulence through continuous replication in poultry populations, live vaccines may also serve as a genetic source contributing to reassortment events. Hence, the development of safer and more efficacious vaccine strategies is critical for improved control of IBDV.

Many factors can contribute to the reassortment process, including vaccine pressure (using different kinds of vaccine such as inactivated vaccines and different attenuated live vaccines), environment (such as temperature, light, humidity, and breeding density), and the immune system of chickens (such as response to the vaccine and environment). In addition, reassortment events may require virus-specific factors such as specific amino acid substitutions in viral proteins. Finally, reassortment may take a long time, and the virus population may require being transferred between chickens or generations of chickens.

In summary, in all eight field isolates investigated here, the segment A sequences of the isolates were identical to vvIBDV, and all segment B sequences were attenuated classical IBDV. This further confirmed that all the isolates used in this study are the natural reassortant IBDV strain and not a mixed population of vvIBDV and attenuated IBDV strains. Therefore, we conclude that all the eight isolates are novel natural reassortant IBDV isolates, which may act as a useful tool to study the replication and pathogenicity of IBDV.

Although segment A of all isolates carries VP2 signatures characteristic of vvIBDV, their actual virulence was not experimentally verified in this study. Since segment B encodes VP1, a key determinant of replication efficiency and pathogenicity, the attenuated-like segment B may alter the in vivo behavior of these reassortants in ways that cannot be inferred from segment A alone.

### 4.3. Recombination Analysis

Genomic segment reassortment among different IBDV strains has been clearly demonstrated in earlier studies [45,112,113,114]. In contrast, there is currently no evidence from India indicating that homologous recombination occurs among circulating IBDV strains. While recombination is relatively common in many positive-sense RNA viruses [116], it is considered uncommon in negative-sense RNA viruses. This rarity is thought to stem from the encapsidation of their genomes within ribonucleoprotein complexes, which limits the availability of the RNA template required for template-switching events [117].

Thirteen potential IBDV recombinations were identified in the present study. In segment A, four potential recombination events were detected, and these recombinations are mainly due to the very virulent nature of the strains. Isolates BGE14, NKL14, and VCN14 were identified as potential recombinants between very virulent and variant strain lineage. Their closest parental strain was identified as T09 (very virulent Bangladesh strain). In segment B, nine potential recombination events were detected, which were mainly the classical virulent strain. Interestingly, among the nine recombination events, eight events had potential recombination between classical virulent and very virulent strains. Their closest parental strain was identified as Cu-1 (classical strain). The isolate RPM14 had recombination between classical virulent strains and attenuated strains. The breakpoints of these two potential recombinants were estimated at almost identical locations, suggesting that they were likely to have originated from the same recombination events.

The study results suggest that there may be a potential recombination between the lineage of segment A very virulent strains and segment A variant strains. Its closest segment A variant parental strain was PBG-98, which is a UK antigenic variant. The genome of segment B shares higher nucleotide and amino acid identity with strain Cu-1 (a classical virulent strain). Based on the above findings, it could be concluded that all the eight isolates might have originated from a classical attenuated strain, and the genome segment A may have recombined with a variant strain.

For all the eight isolates, it is possible that homologous recombination may have occurred between live vaccines (since most of the live vaccines used in India comprise cell culture-adapted attenuated IBDV strains) and wild-type IBDV strains to produce very virulent isolates. Flock-wide mass vaccination may also elevate the likelihood of generating chimeric viruses through recombination or reassortment events. Although live vaccines are generally effective, concerns persist regarding their potential to revert to virulence or to contribute to the emergence of recombinant viruses [118]. Similar issues have been observed in poliovirus, where more than half of the strains associated with vaccine-derived paralytic poliomyelitis were found to be recombinants [119]. While most of these recombinant polioviruses did not exhibit markedly different characteristics, a few showed altered virulence [120,121]. For IBDV, pathogenicity and tissue tropism are governed mainly by specific residues in VP2 and, to some extent, by VP1 [28,43]. Vaccine strains may potentially acquire these virulence-associated determinants through intra-serotype recombination with circulating wild-type viruses. Therefore, although it cannot be ruled out, the emergence of chimeric viruses with significantly modified phenotypes through recombination remains a theoretical but important concern. In IBDV, reassortment—the exchange of entire genome segments—is a well-established evolutionary mechanism, whereas true homologous recombination within a segment is considered rare in dsRNA viruses due to their capsid-restricted replication.

## 5. Conclusions

This study represents a detailed investigation of actively circulating strains of IBDV from Southern India utilizing complete genome sequencing and analysis of multiple field isolates. Analysis of the whole genome sequences of the isolates revealed that segment A belongs to vvIBDV strains and segment B belongs to classical attenuated strains in all the isolates. This indicates the occurrence of possible natural reassortants. This study suggests that the molecular characterization of IBDV can be performed using the partial sequencing of both segments; segment A (hypervariable region) and segment B (5′ N-terminal first 300 amino acids) should be used instead of either one of the segments alone. This study also addresses the need for the development of a recombinant vaccine for control of IBDV and reinforces that continuous surveillance of Serotypes I and II are essential for understanding the molecular epidemiology of IBDV in India.

## Figures and Tables

**Table 1:** Details of nucleotide length and ORF positions in segment A and B.

IBDV Isolates	Segment A					Segment B				
	Total nt Length	Gene	Position	No. of nts	No. of aas	Total nt Length	Gene	Position	No. of nts	No. of aas
BGE15	3265	VP5	89–538	450	150	2830	VP1	115–2751	2637	879
		Polyprotein	135–3173	3039	1013					
BGE14	3260	VP5	97–534	438	146	2838	VP1	123–2759	2637	879
		Polyprotein	131–3169	3039	1013					
EDE14	3261	VP5	97–534	438	146	2827	VP1	112–2751	2640	880
		Polyprotein	131–3154	3024	1008					
MDI14	3259	VP5	96–533	438	146	2833	VP1	112–2757	2646	882
		Polyprotein	130–3168	3039	1013					
NKL14	3260	VP5	97–534	438	146	2827	VP1	112–2751	2640	880
		Polyprotein	131–3169	3039	1013					
VCN14	3260	VP5	97–534	438	146	2830	VP1	115–2754	2640	880
		Polyprotein	131–3169	3039	1013					
RPM14	3261	VP5	97–534	438	146	2827	VP1	112–2751	2640	880
		Polyprotein	131–3154	3024	1008					
THI14	3260	VP5	97–534	438	146	2827	VP1	112–2751	2640	880
		Polyprotein	131–3169	3039	1013					

**Table 2:** Details of amino acid variations in segment A and B.

IBDV Isolates	Segment A						Segment B
	ORF1	ORF2					
	VP5	VP2 (1–441)	VP2 (442–512)	VP4 (513–755)	VP3 (756–1013)	Total	VP1
BGE15	6	3	2	0	3	11	18
BGE14	3	3	2	0	3	14	19
EDE14	5	3	2	1	17	28	22
MDI14	5	3	2	1	4	15	35
NKL14	5	3	2	3	3	16	21
VCN14	17	11	2	8	5	43	21
RPM14	4	3	2	1	16	26	23
THI14	5	3	2	6	7	23	21

**Table 3:** Summary of unique recombination events identified by RDPv.4.16 (RDP4).

Recombination Event Number	Breakpoint Positions in Recombinant Sequence		Recombinant Sequence(s)	Parental Sequence(s)		Score for the Six Detection Methods in RDP4					
	Begin	End		Minor	Major	RDP	GENECONV	Boots can	Maxchi	Chimaera	SiSscan
**Segment A**											
1	1427	2073	BGE14	D00868 (PBG-98)	AF362776 (BD-3/99)	2.62 × 10^−7^	1.38 × 10^−5^	1.25 × 10^−2^	7.59 × 10^−7^	7.30 × 10^−7^	3.43 × 10^−9^
2	785	3163	BGE14	D00868 (PBG-98)	AY099456	1.74 × 10^−3^	1.30 × 10^−5^	1.27 × 10^−3^	2.97 × 10^−2^	1.90 × 10^−2^	8.29 × 10^−5^
1	1427	2798	NKL14	AF165149	AF362776 (BD-3/99)	5.23 × 10^−10^	1.93 × 10^−8^	2.14 × 10^−8^	2.32 × 10^−13^	1.44 × 10^−12^	5.70 × 10^−18^
1	743	1324	VCN14	AF36276 (BD-3/99)	AF165149	4.48 × 10^−13^	2.14 × 10^−12^	4.76 × 10^−12^	6.77 × 10^−12^	2.64 × 10^−12^	4.96 × 10^−15^
**Segment B**											
1	1152	1592	BGE15	AF362770	AF362775	4.59 × 10^−23^	3.89 × 10^−22^	6.27 × 10^−16^	8.27 × 10^−14^	1.43 × 10^−11^	5.68 × 10^−11^
1	2220	2648	EDE14	D49707	AF362775	3.16 × 10^−18^	5.02 × 10^−16^	8.92 × 10^−7^	9.93 × 10^−11^	5.14 × 10^−10^	5.64 × 10^−12^
2	1302	1550	EDE14	D49707	AF362775	3.28 × 10^−3^	1.32 × 10^−12^	2.95 × 10^−11^	4.27 × 10^−9^	6.96 × 10^−8^	1.95 × 10^−8^
1	1051	1744	MDI14	AF527040	AF362775	6.38 × 10^−16^	4.44 × 10^−14^	1.12 × 10^−13^	1.84 × 10^−18^	2.07 × 10^−4^	5.98 × 10^−12^
1	2211	2656	NKL14	DQ927041	AF362775	2.14 × 10^−28^	1.42 × 10^−26^	1.52 × 10^−20^	2.00 × 10^−11^	3.25 × 10^−11^	4.70 × 10^−14^
1	2236	2656	RPM14	D49707	AF362775	1.33 × 10^−15^	3.28 × 10^−13^	6.41 × 10^−7^	1.51 × 10^−9^	2.72 × 10^−9^	1.37 × 10^−10^
2	1236	1550	RPM14	DQ927041	JF811921	5.49 × 10^−18^	1.09 × 10^−13^	0.010485	2.28 × 10^−9^	9.19 × 10^−10^	7.35 × 10^−11^
1	1169	1552	THI14	AF527040	AF362775	8.63 × 10^−5^	1.17 × 10^−6^	5.78 × 10^−10^	9.24 × 10^−11^	2.88 × 10^−9^	1.75 × 10^−6^
1	2211	2656	VCN14	D49707	AF362775	6.99 × 10^−20^	3.08 × 10^−10^	1.31 × 10^−6^	6.92 × 10^−10^	4.24 × 10^−9^	1.56 × 10^−8^

Minor Parent = Parent contributing the smaller fraction of sequence. Major Parent = Parent contributing the larger fraction of sequence.

## Data Availability

The data presented in the study were deposited to the National Centre for Biotechnology Information repository with the following accession numbers [BGE15-KT870148 (Segment A) and KT870149 (Segment B); BGE14-KT884452 (Segment A) and KT884453 (Segment B); EDE14-KU558697 (Segment A) and KU558698 (Segment B); MDI14-KU558699 (Segment A) and KU558700 (Segment B); NKL14-KU578098 (Segment A) and KU578099 (Segment B); VCN14-KU578100 (Segment A) and KU578101 (Segment B); RPM14-KU578102 (Segment A) and KU578103 (Segment B); THI14-KU578104 (Segment A) and KU578105 (Segment B)].

## Supplementary Materials

The following supporting information can be downloaded at: https://www.mdpi.com/article/10.3390/pathogens15010026/s1, File S1: RT-PCR Primer sequence, time and temperature combinations used for amplification of segment A and B of IBDV.

## Abbreviations

The following abbreviations are used in this manuscript:

IBDV	Infectious Bursal Disease Virus
RT-PCR	Reverse Transcriptase–Polymerase Chain Reaction
CAM	Chorio Allantoic Membrane
vvIBDV	Very virulent IBDV
cvIBDV	Classical virulent IBDV
aa	Amino Acid
nt	Nucleotide
