# Supplementary material for: Deciphering the Natural Reassortment Dynamics of Infectious Bursal Disease Virus, Isolated from Field Outbreaks in Southern India, Through Complete Genome Sequencing
Source: Pathogens. 2025 Dec 24;15(1):26. doi: 10.3390/pathogens15010026 (PMC12845039; doi:10.3390/pathogens15010026)
Supplement: Supplementary file 1 [file pathogens-15-00026-s001.zip › pathogens-3997580-supplementary.pdf]

Supplementary file 1. RT-PCR Primer sequence, time and temperature combinations used for amplification of segment A and B of IBDV

Primer sequences used for amplification of segment A and B of IBDV genome

S.No.	Names of Primers	Primer sequences (5′-3′)	Nucleotide position of primers
**Primers for amplification of 5′ end of segment A**			
1.	IBDVSegA 5′FP	GGA TAC GAT CGG TCT GAC CCC	1-21
2.	IBDVSegA 5′RP	CCG GTA GGT TCT GGG CAG TC	391-410
**Primers for amplification of other regions of segment A**			
3.	IBDVSegA 55FP	CTT GTT CCA GGC TGG AAC TCC TCC	55-79
4.	IBDVSegA 55RP	GCC TGT GCC TCA TCG CCC AG	1574-1594
5.	IBDVSegA 1507FP	TGG TCT CCA CAT TGT TCC CAC C	1507-1529
6.	IBDVSegA 1507RP	GCC GAA TTG GTG TCC ACA CCT	3205-3226
**Internal sequencing primers used for amplification of segment A**			
7.	IBDVSegA 320FP	CCC TGG CTC AAT TGT GGG TGC	320-341
8.	IBDVSegA 320RP	TGA GAA CAG TGT GAT TGT TAC CCC	800-824
9.	IBDVSegA 2223FP	CAC CGA CTT GGC CTT AAG TTG GC	2223-2246
10.	IBDVSegA 2223RP	GAT GCC CAT GGT CTG CAT CTT CTT	2712-2736
**Primers for amplification of 3′ end of segment A**			
11.	IBDVSegA 3′FP	AGG CGG GCT CCA CCA AAG	3062-3079
12.	IBDVSegA 3′RP	GGG GAC CCG CGA ACG GAT C	3242-3260
**Primers for amplification of 5′ end of segment B**			
13.	IBDVSegB 5′FP	GGA TAC GAT GGG TCT GAC CCT C	1-22
14.	IBDVSegB 5′RP	TGC CGA ACG CTG CTG AGA T	151-169
**Primers for amplification of other regions of segment B**			
15.	IBDVSegB 40FP	CGT GGC TAC TAG GGG CGA TA	40-59
16.	IBDVSegB 40RP	TGC GCG ATG CTC TCA AAA G	704-722
17.	IBDVSegB 651FP	GGA GGT CGC CAC TGG AAG A	651-669
18.	IBDVSegB 651RP	CGG GGC CAA TAT CCA CTC	1270-1287
19.	IBDVSegB 1112FP	CCC GGA ACA TAT GGT CAG CTC	1112-1132
20.	IBDVSegB 1112RP	TCC CGA GAT CTT TGC TGT ATG	1838-1858
21.	IBDVSegB 1639FP	CCC AGA CCA GAC AGC GAG GA	1639-1658
22.	IBDVSegB 1639RP	ACG GCT TCT TGC TGT GGC TA	2321-2340
23.	IBDVSegB 2211FP	GCC CCC AAA TGT CAA CAG A	2211-2229
24.	IBDVSegB 2211RP	CGG GGT CTG GGA TTA GTG TC	2778- 2797
**Primers for amplification of 3′ end of segment B**			
25.	IBDVSegB 3′FP	CCT GCC CGC CAA GAG AGC	2604-2621
26.	IBDVSegB 3′RP	GGG GGC CCC CGC AGG CGA A	2807-2827

RT-PCR reaction conditions for amplification of different regions of IBDV genome

Name of Primers	Initial denaturation	Cycle conditions			No of cycles	Final extension	Products size
		Denaturation	Annealing	Extension			
**RT-PCR amplification of VP2 region**							
IBDVP2 743FP/RP	94°C for 2 min	94°C for 1.50 min	53 °C for 1min	72°C for 1 min	35	72°C for 7 min	743bp
**RT-PCR amplification of VP1 region**							
IBDVP1 749FP/RP	94°C for 2 min	94°C for 1.50 min	59 °C for 1min	72°C for 1 min	35	72°C for 7 min	749bp
**RT-PCR amplification of 5′ end of segment A**							
IBDVSegA 5′FP/RP	94°C for 2 min	94°C for 1.50 min	63 °C for 30 sec	72°C for 1 min	35	72°C for 7 min	410bp
**Long Range PCR amplification of Segment A**							
IBDVSegA 55FP/RP	98 °C for 10 sec	94°C for 20 sec	56 °C for 1 min	68 °C for 2 min	35	72°C for 10min	1539bp
IBDVSegA 1507FP/RP	98 °C for 10 sec	94°C for 20 sec	55 °C for 1 min	68 °C for 2 min	35	72°C for 10min	1719bp
**RT-PCR amplification of Segment A**							
IBDVSegA 1183FP/RP	94°C for 2 min	94°C for 1.50 min	61 °C for 30 sec	72°C for 1 min	35	72°C for 7 min	983bp
**Internal primers used for sequencing of segment A**							
IBDVSegA 320FP/RP	94°C for 2 min	94°C for 1.50 min	57 °C for 30 sec	72°C for 1 min	35	72°C for 7 min	520bp
IBDVSegA 2223FP/RP	94°C for 2 min	94°C for 1.50 min	65 °C for 30 sec	72°C for 1 min	35	72°C for 7 min	513bp
IBDVSegA 3′FP/RP	94°C for 2 min	94°C for 1.50 min	67 °C for 30 sec	72°C for 1 min	35	72°C for 7 min	198bp
**RT-PCR amplification of 5′ end of segment B**							
IBDVSegB 5′FP/RP	94°C for 2 min	94°C for 1.50 min	54 °C for 30 sec	72°C for 1 min	35	72°C for 7 min	169bp
**RT-PCR amplification of Segment B**							
IBDVSegB 40FP/RP	94°C for 2 min	94°C for 1.50 min	60 °C for 1 min	72°C for 1 min	35	72°C for 7 min	683bp
IBDVSegB 651FP/RP	94°C for 2 min	94°C for 1.50 min	62 °C for 1 min	72°C for 1 min	35	72°C for 7 min	637bp
IBDVSegB 1112FP/RP	94°C for 2 min	94°C for 1.50 min	60 °C for 1 min	72°C for 1 min	35	72°C for 7 min	753bp
IBDVSegB 1639FP/RP	94°C for 2 min	94°C for 1.50 min	63 °C for 1 min	72°C for 1 min	35	72°C for 7 min	703bp
IBDVSegB 2211FP/RP	94°C for 2 min	94°C for 1.50 min	60 °C for 1 min	72°C for 1 min	35	72°C for 7 min	587bp
**RT-PCR amplification of 3′ end of segment B**							
IBDVSegB 3′FP/RP	94°C for 2 min	94°C for 1.50 min	62 °C for 30 sec	72°C for 1 min	35	72°C for 7 min	224bp

.

Details of VP5 amino acid variations

S.No	AA Position	vvIBDV	cvIBDV	Variant IBDV	Attenuated IBDV	BGE14	BGE15	EDE14	MDI14	NKL14	RPM14	THI14	VCN14
1.	3	S	S	S	S	S	S	S	S	S	S	S	T
2.	7	T	T	T	T	T	T	T	T	T	T	T	R
3.	8	N	N	N	N	N	N	N	N	N	N	N	K
4.	9	D	D	D	D	D	D	D	D	D	D	D	H
5.	11	S	S	S	S	S	S	S	S	S	S	S	G
6.	17	R	R	R	R	R	G	R	R	R	R	R	I
7.	19	N	N	N	N	N	N	N	N	N	N	N	M
8.	33	N	N	N	N	N	N	N	N	N	N	N	G
9.	34	N	N	N	N	N	N	N	N	N	N	N	H
10.	39	H	H	H	H	H	H	H	H	H	H	H	R
11.	43	H	H	H	H	H	H	H	H	H	H	H	L
12.	45	R	G	G	G	R	R	G	R	G	R	R	G
13.	47	A	A	A	A	A	T	A	A	A	A	A	A
14.	57	Q	Q	Q	Q	Q	Q	Q	Q	Q	Q	Q	R
15.	74	L/F	I	I	I	I	F	I	I	I	I	F	I
16.	78	N	N	N	N	N	N	N	N	N	N	N	D
17.	87	E	E	E	E/G	E	E	E	E	E	E	E	E
18.	100	C	C	C	C	C	C	C	C	C	C	G	C
19.	112	A	T/A	A	A	A	A	A	A	A	A	A	A
20.	118	H	H	H	H	H	N	N	N	H	N	N	H
21.	120	E	E	K	E	E	E	E	E	E	E	E	E
22.	125	P/S	P	P	S	H	N	N	N	H	N	N	H
23.	133	W	R	R	R	R	R	W	R	R	W	R	R
24.	134	H	H	H	H	H	N	N	N	N	N	N	H

References used for comparison: UK661 for vvIBDV, 52/70 F for cvIBDV, Variant E for variant and 903/78 for Attenuated strain

Details of amino acid substitutions at VP2 region

S.No	AA Position	vvIBDV	cvIBDV	Variant IBDV	Attenuated IBDV	BGE14	BGE15	EDE14	MDI14	NKL14	RPM14	THI14	VCN14
1.	6	D	D	D	D	D	D	D	D	D	D	D	Y
2.	7	Q	Q	Q	Q	Q	Q	Q	Q	Q	Q	Q	P
3.	8	T	T	T	T	T	T	T	T	T	T	T	C
4.	16	R	R	R	R	R	R	R	R	R	R	R	L
5.	18	L	L	L	L	L	L	L	L	L	L	L	H
6.	22	T	T	T	T	T	T	T	T	T	T	T	A
7.	28	I	I	I	I	I	I	I	I	I	I	I	V
8.	32	T	T	T	T	T	T	T	T	T	T	T	S
9.	46	N	N	N	N	N	N	N	N	N	N	N	D
10.	212	D	D	D	D	D	D	D	D	N	D	D	D
11.	213	D	D	N	D	D	D	D	D	D	D	D	D
12.	222	A	P	T	P	A	A	A	A	A	A	A	A
13.	240	L	L	L	L	F	F	F	F	L	F	F	L
14.	242	I	I	V	V	I	I	I	I	I	I	I	I
15.	249	Q	Q	K	R	Q	Q	Q	Q	Q	Q	Q	Q
16.	253	Q	Q	Q	H	Q	Q	Q	Q	Q	Q	Q	Q
17.	254	G	G	S	S	G	G	G	G	G	G	G	G
18.	255	L	L	L	L	L	L	L	L	L	L	L	L
19.	256	I	V	V	V	I	I	I	I	I	I	I	I
20.	257	L	L	L	L	L	L	L	L	L	L	L	L
21.	262	Y	Y	C	Y	Y	Y	Y	Y	Y	Y	Y	Y
22.	263	L	L/F	L	L	L	L	L	L	L	L	L	L
23.	269	T	T	T/S	T	T	T	T	T	T	T	T	T
24.	270	A	A/T	A	T	T	T	T	T	T	T	T	T
25.	279	D	D	N	N	D	D	D	D	D	D	D	D
26.	280	N	N	N	N	N	N	N	N	N	N	N	N
27.	284	A	A	A	T	A	A	A	A	A	A	A	A
28.	286	T	T	I	T	T	T	T	T	T	T	T	T

S.No	AA Position	vvIBDV	cvIBDV	Variant IBDV	Attenuated IBDV	BGE14	BGE15	EDE14	MDI14	NKL14	RPM14	THI14	VCN14

29.	290	M/L	M	M	M/L	M	M	M	M	M	M	M	M
30.	294	I	L	L	L	V	V	V	V	V	V	V	V
31.	297	P	P	P	P/S	P	P	P	P	P	P	P	P
32.	298	T/I	T	T	T	T	T	T	T	T	T	T	T
33.	299	S	N	N	N	S	S	S	S	S	S	S	S
34.	300	E/A/Q	E	E	E	A	A	A	A	A	A	A	A
35.	312	I	I/V	I	I	I	I	I	I	I	I	I	I
36.	318	G	G	D	G	G	G	G	G	G	G	G	G
37.	330	S/R	S	S	S	S	S	S	S	S	S	S	S
38.	451	L	I	I	I	I	I	I	I	I	I	I	I
39.	469	L	L	V	L	L	L	L	L	L	L	L	L
40.	481	L	L	R	C	C	C	C	C	C	C	C	C

References used for comparison: UK661 for vvIBDV, 52/70 F for cvIBDV, Variant E for variant and 903/78 for attenuated strain

Details of amino acid variations in VP3 region

S.No	AA Position	vvIBDV	cvIBDV	Variant IBDV	Attenuated IBDV	BGE14	BGE15	EDE14	MDI14	NKL14	RPM14	THI14	VCN14
1.	822	P	P	L/P	P	P	P	P	P	P	P	P	P
2.	849	G	E	G/E	E	E	E	E	E	E	E	E	E
3.	858	I	I	I	I	I	I	I	I	I	I	I	T
4.	863	E	E	E	E	E	E	E	E	E	E	Q	E
5.	868	Y	Y	Y	Y	Y	Y	Y	Y	Y	Y	N	Y
6.	872	P	P	Q	P	P	P	P	P	P	P	P	P
7.	877	L	L	F	L	L	L	L	L	L	L	L	L
8.	879	G	G	R	G	G	G	G	G	G	G	G	G
9.	882	G	G	R	G	G	G	G	G	G	G	G	G
10.	886	G	G/A	G	G	G	G	G	G	G	G	G	G
11.	903	D/E	D	Y	D	D	D	D	D	D	D	D	D
12.	919	G/E	E	E	E	G	E	E	E	E	E	E	E
13.	922	L	L	L	L	L	L	L	L	L	L	L	Q
14.	923	R	R/K	R	R	R	R	R	R	R	R	R	R
15.	929	Y	Y	Y	Y	Y	Y	Y	F	Y	Y	F	Y
16.	933	G	G	V	G	G	G	G	G	G	G	G	G
17.	938	P	P	L	P	P	P	P	P	P	P	P	P
18.	951	V	I	I	I	I	I	I	I	I	I	I	I
19.	972	M/L	M	M	M	M	M	M	M	M	M	M	M
20.	990	V	A	A	A	A	A	G	A	A	G	A	A
21.	991	P	P	P	P	P	P	S	P	P	S	P	P
22.	992	T	T	T	T	T	T	N	T	T	N	T	T
23.	993	Q	Q	Q	Q	Q	Q	T	Q	Q	T	L	Q
24.	994	R	R	R	R	R	R	E	R	R	E	R	R
25.	995	P	P	P	P	P	P	T	P	P	T	P	P
26.	1007	S	S	S	S	S	S	L	S	S	L	S	S
27.	1009	E	E	E	E	E	E	Q	E	E	Q	E	E
28.	1010	D	D	D	D	D	D	G	D	D	G	D	D
29.	1011	L	L	L	L	L	L	C	L	L	C	L	L
30.	1012	E	E	E	E	E	E	L	E	E	L	E	E

References used for comparison: UK661 for vvIBDV, 52/70 F-cvIBDV, Variant E- Variant and 903/78-Attenuated strain

Details of amino acid variations in VP4 region

S.No	AA Position	vvIBD	cvIBD	Variant IBD	Attenuated IBD	BGE14	BGE15	EDE14	MDI14	NKL14	RPM14	THI14	VCN14
1.	527	P	P	P	P	P	P	T	P	P	P	P	P
2.	541	I/V	V	I	V	V	I	I	I	V	I	I	V
3.	577	S	S	N	S	S	S	S	S	S	S	S	S
4.	579	M	M/I	M	M	M	M	M	M	M	M	M	M
5.	624	V/D	D	D	D	D	D	D	D	D	D	D	D
6.	632	G/D	D	D	D	D	D	D	D	D	D	D	D
7.	642	K/N	K	K	K	K	K	N	R	K	K	R	K
8.	651	S/N	N	N	N	N	N	N	N	N	N	N	N
9.	676	A	A	A	A	A	A	A	A	A	A	T	A
10.	680	Y/C	F/C	C	C	Y	Y	Y	Y	C	Y	Y	S
11.	684	E	E	E	E	E	E	E	E	E	E	E	G
12.	685	N/K/S	K	K	K	N	N	N	N	K	N	N	K
13.	686	V	I/V	I	V	V	V	V	V	V	V	G	V
14.	687	S	S	S	S	S	S	S	S	S	S	V	S
15.	688	F	F	F	F	F	F	F	F	F	F	S	F
16.	690	S	S	S	S	S	S	S	S	S	S	G	S
17.	702	K	K	K	K/R	K	K	K	K	R	K	K	K
18.	715	S	P	P	P	S	S	S	S	P	S	S	P
19.	751	D	H	H	H	D	D	D	D	H	H	D	H

References used for comparison: UK661 for vvIBDV, 52/70 F for cvIBDV, Variant E for variant and 903/78 for attenuated strain

Details of amino acid variations in VP1 Protein

S.No	AA Position	vvIBDV	cvIBDV	Variant IBDV	Attenuated IBDV	BGE14	BGE15	EDE14	MDI14	NKL14	RPM14	THI14	VCN14
1.	4	V	I	I	V/I	I	I	I	I	I	I	I	I
2.	12	S/Q	T	S	S	S	S	S	S	S	S	S	S
3.	13	K	K	K	T	T	T	T	T	T	T	T	T
4.	61	I	V	V	V	V	V	V	V	V	V	V	V
5.	69	E/D	E	E	E	E	E	E	E	E	E	E	E
6.	91	P/A	A	A	A	A	A	A	A	A	A	A	A
7.	92	V	A	V	V	V	V	V	V	V	V	V	V
8.	104	Q	Q	H	Q	Q	Q	Q	Q	Q	Q	Q	Q
9.	136	Y	F	Y	Y	Y	Y	Y	Y	Y	Y	Y	Y
10.	145	T	N	N	N	N	N	N	N	N	N	N	N
11.	146	D	E	E	E	E	E	E	E	E	E	E	E
12.	147	N	G	G	G	G	G	G	G	G	G	G	G
13.	150	D	E	D	D	D	D	D	D	D	D	D	D
14.	158	N	N	N	N	N	N	T	N	N	N	N	N
15.	170	M	M	I	M	M	M	M	M	M	M	M	M
16.	204	Q	H	Q	Q	Q	Q	Q	Q	Q	Q	Q	Q
17.	205	L	V	L	L	L	L	L	L	L	L	L	L
18.	216	G	G	G	G	G	G	G	G	G	G	C	G
19.	217	E	E	E	E	E	E	E	E	E	E	K	E
20.	242	E	D	D	D	D	D	D	D	D	D	D	D
21.	287	A	T	T	T	T	T	T	T	T	T	T	T
22.	325	Y	Y	Y	Y	Y	Y	Y	Y	Y	Y	C	Y
23.	330	C	W	W	W	W	W	W	W	W	W	W	W
24.	331	C	L	L	L	L	L	L	L	L	L	L	L
25.	333	K	K	K	K	K	K	K	N	K	K	K	K
26.	337	K	I	I	I	I	I	I	I	I	I	I	I
27.	341	Q	P	P	P	P	P	P	P	P	P	P	P
28.	343	S	P	P	P	P	P	P	Q	P	P	P	P
29.	390	M	L	L	L	L	M	L	M	L	L	M	L
30.	393	D/V	E	E	E	E	D	E	D	E	E	D	E
31.	444	N	N	N	N	N	D	N	N	N	N	D	N
32.	484	S	S	M	S	S	S	S	S	S	S	S	S

33.	495	L	L	L	L	L	L	L	L	L	L	F	L
34.	496	L	L	L	L	L	L	V	L	L	V	L	L
35.	508	K	R	R	R	R	K	K	K	R	K	K	R
36.	511	S	R	R	R	R	S	R	S	R	R	R	R
37.	515	E	E	D	E	E	E	E	E	E	E	E	E
38.	546	P	P	P	L	L	L	L	P	L	L	L	L
39.	548	A	A	A	A	A	A	A	K	A	A	A	A
40.	549	Q	Q	Q	Q	Q	Q	Q	G	Q	Q	Q	Q
41.	550	P	P	P	P	P	P	P	T	P	P	P	P
42.	551	G	G	G	G	G	G	G	R	G	G	G	G
43.	552	Y	Y	Y	Y	Y	Y	Y	V	Y	Y	Y	Y
44.	553	L	L	L	L	L	L	L	P	L	L	L	L
45.	554	S	S	S	S	S	S	S	E	S	S	S	S
46.	555	G	G	G	G	G	G	G	W	G	G	G	G
47.	556	G	G	G	G	G	G	G	D	G	G	G	G
48.	557	V	V	V	V	V	V	V	W	V	V	V	V
49.	558	E	E	E	E	E	E	E	N	E	E	E	E
50.	559	P	P	P	P	P	P	P	Q	P	P	P	P
51.	560	E	E	E	E	E	E	E	S	E	E	E	E
52.	561	Q	Q	Q	Q	Q	Q	Q	N	Q	Q	Q	Q
53.	562	P	S	S	S	S	S	S	P	S	S	S	S
54.	563	S	S	S	S	S	S	S	A	S	S	S	S
55.	565	T	T	T	T	T	T	T	R	T	T	T	T
56.	566	V	V	V	V	V	V	V	E	V	V	V	V
57.	682	K	K	R	K	K	K	K	K	K	K	K	K
58.	685	V	V	I	V	V	V	V	V	V	V	V	V
59.	687	P	S	S	S	S	S	S	S	S	S	S	S
60.	695	P	K	K	K	K	K	K	K	K	K	K	K
61.	718	N	N	S	N	N	N	N	N	N	N	N	N
62.	751	M/K	K	K	K	K	K	K	K	K	K	K	K
63.	825	H/N	N	N	N	N	N	N	N	N	N	N	N
64.	859	T	T	T	T	T	T	T	T	T	T	T	A

References used for comparison: UK661 for vvIBDV, 52/70 F for cvIBDV, Variant E for variant and 903/78 for attenuated strain
